# Effects of Mind–Body Exercise on Pulmonary Function and Functional Exercise Capacity in Older Adults with COPD: A Three-Level Meta-Analysis

**DOI:** 10.3390/life16091444

**Published:** 2026-08-30

**Authors:** Weiping Du, Songnian Han, Xiaodan Niu, Aiping Chi, Jianhua Zhou

**Affiliations:** 1 School of Physical Education, Ningxia Normal University, Guyuan 756099, China; 82015006@nxnu.edu.cn (W.D.); 82023043@nxnu.edu.cn (S.H.); 2 School of Physical Education, Shaanxi Normal University, Xi’an 710119, China; 3 School of Teacher Development, Shaanxi Normal University, Xi’an 710119, China; 19591386242@163.com; 4 School of Marxism, Qinghai Minzu University, Xining 810007, China

**Keywords:** chronic obstructive pulmonary disease, mind–body exercise, pulmonary function, six-minute walk distance, three-level meta-analysis

## Abstract

**Background/Objectives**: Mind–body exercise may improve pulmonary function and exercise capacity in older adults with chronic obstructive pulmonary disease (COPD), but its overall effects and potential variation by exercise modality and intervention duration remain uncertain. This study evaluated its effects on pulmonary function and six-minute walk distance (6MWD) in COPD samples with a mean age of ≥60 years. **Methods**: PubMed, Web of Science Core Collection, Embase, the Cochrane Library, CNKI, and Wanfang Data were searched from inception to June 2026. Randomized controlled trials comparing mind–body exercise with control conditions were included. Pulmonary function was analyzed using a three-level random-effects meta-analysis with study-clustered CR2 robust inference; 6MWD was analyzed using two-level random-effects models with Knapp–Hartung inference. Hedges’ g and raw mean differences (MDs) were reported. Exploratory subgroup and meta-regression analyses examined exercise modality and intervention duration, with Holm correction for multiple comparisons. The review was retrospectively registered with INPLASY (INPLASY202670087). **Results**: Thirty-five independent studies involving 2955 participants were included. Mind–body exercise improved overall pulmonary function (Hedges’ g = 0.62, 95% CI 0.40–0.83, *p* < 0.001), with substantial heterogeneity (I^2^ = 91.0%; 95% prediction interval [PI] −0.61 to 1.84). Pooled effects for FEV_1_, FVC, and FEV_1_/FVC were g = 0.71, 0.52, and 0.53, respectively. For 6MWD, g = 0.78 (95% CI 0.57–0.99), corresponding to an MD of 34.07 m (95% CI 25.88–42.25; 95% PI 7.53–60.60). After Holm correction, neither exercise modality nor intervention duration showed a significant moderating effect, and no robust linear or quadratic duration association was identified. **Conclusions**: Mind–body exercise may improve pulmonary function and functional exercise capacity in older adults with COPD. However, benefits may vary across settings, and current evidence is insufficient to identify a superior modality or optimal intervention duration. Mind–body exercise may be considered an adjunctive or optional component of comprehensive pulmonary rehabilitation.

## 1. Introduction

Chronic obstructive pulmonary disease (COPD) is characterized by chronic respiratory symptoms and persistent airflow limitations. Acute exacerbations further increase the disease burden, and COPD remains a major global cause of mortality and disability, with its burden increasing markedly with age [1,2]. With advancing age, the cumulative effects of comorbidities and skeletal muscle dysfunction make older adults with COPD particularly susceptible to reduced exercise tolerance and limitations in activities of daily living [3,4]. Forced expiratory volume in one second (FEV_1_), forced vital capacity (FVC), and the FEV_1_/FVC ratio reflect different dimensions of pulmonary function [5], whereas the six-minute walk distance (6MWD) provides an integrated measure of functional exercise capacity [2]. Accordingly, pulmonary function and 6MWD provide complementary information for evaluating rehabilitation outcomes.

Pulmonary rehabilitation is a key non-pharmacological intervention for patients with stable COPD and for those recovering from hospitalization for acute exacerbation. Comprehensive pulmonary rehabilitation typically includes individualized assessment, exercise training, education, and behavioral support [6,7]. These interventions can improve exercise capacity, dyspnea, and health-related quality of life; however, their implementation may be constrained by limited program accessibility, transportation barriers, and service capacity [7]. Mind–body exercises, which require relatively little equipment and can be practiced in community or home settings, may therefore represent a useful adjunct to comprehensive pulmonary rehabilitation [8].

Mind–body exercise generally integrates low- to moderate-intensity physical activity with breathing regulation and attentional control [9]. However, the training characteristics of different modalities are not identical. Liuzijue emphasizes vocalization and breathing control [10]; Baduanjin involves repeated trunk and limb movements, postural control, and coordinated movement patterns; and Tai Chi incorporates continuous movement sequences, weight shifting, balance control, breathing, and attentional regulation [11,12]. Thus, although these three exercise modalities can reasonably be considered within a broader mind–body exercise framework when evaluating their overall rehabilitative value, they should not be assumed to impose identical biomechanical demands, respiratory training components, or physiological pathways. Exercise modality should therefore be considered a potential effect modifier.

From a physiological perspective, mind–body exercise may influence COPD-related outcomes through several distinct but interrelated pathways. Slow or prolonged expiration may improve breathing control and respiratory muscle function, whereas weight-bearing and limb movements may enhance peripheral skeletal muscle function and exercise tolerance, thereby exerting a more direct influence on functional exercise capacity [4,10]. In addition, the integration of breathing regulation and attentional control may influence dyspnea management, self-efficacy, and participation in physical activity [11]. These pathways should, however, be regarded as plausible mechanistic hypotheses rather than established mediating mechanisms.

Previous systematic reviews and meta-analyses suggest that mind–body exercise may improve pulmonary function and exercise capacity in patients with COPD; however, the available evidence has generally included broad age ranges, different exercise modalities and intervention doses, and multiple pulmonary function outcomes, with limited synthesis specifically focused on older adults with COPD [9,12]. At the same time, individual randomized controlled trials frequently report several pulmonary function outcomes, such as FEV_1_, FVC, and FEV_1_/FVC. Selecting only one outcome would discard potentially relevant information, whereas treating multiple effect sizes from the same study as statistically independent may underestimate standard errors and overstate precision. Three-level meta-analysis allows multiple pulmonary function outcomes to be retained while accounting for the dependency among effect sizes within the same study, making it particularly suitable for this data structure [13,14].

Against this clinical and methodological background, the present study included randomized controlled trials in which the study sample had a mean age of ≥60 years and systematically evaluated the effects of mind–body exercise, compared with control conditions, on pulmonary function and 6MWD. A three-level random-effects model was used to analyze multiple pulmonary function outcomes, and pulmonary function outcome type, exercise modality, and intervention duration were further examined as potential effect modifiers. We formulated two directional hypotheses: first, that mind–body exercise would, on average, improve pulmonary function and 6MWD; and second, because exercise training may act more directly on peripheral muscle deconditioning and functional exercise capacity, positive effects on 6MWD would be more consistent across studies, whereas effects on pulmonary function would exhibit greater heterogeneity. Given the limited availability of head-to-head comparisons and robust dose–response evidence, we did not prespecify a hierarchy among exercise modalities or an optimal intervention duration. Analyses of exercise modality and both linear and quadratic associations with intervention duration were therefore treated as exploratory effect-modification analyses.

## 2. Materials and Methods

### 2.1. Study Design and Registration

This systematic review and meta-analysis was reported in accordance with the Preferred Reporting Items for Systematic Reviews and Meta-Analyses (PRISMA) 2020 statement [15] and conducted with reference to the Cochrane Handbook for Systematic Reviews of Interventions [16]. The review was retrospectively registered with INPLASY (Number: INPLASY202670087; https://doi.org/10.37766/inplasy2026.7.0087).

### 2.2. Literature Search

A systematic search was conducted in PubMed, Web of Science Core Collection, Embase, the Cochrane Library, China National Knowledge Infrastructure (CNKI), and Wanfang Data from database inception to June 2026.

For the English-language databases, the search strategies primarily combined terms related to COPD, mind–body exercise, and randomized controlled trials. For CNKI and Wanfang Data, broader searches combining COPD- and mind–body exercise-related terms were used, and eligibility with respect to randomized study design was subsequently determined during study screening.

The complete search strategies and search yields for each database are provided in Supplementary Table S1.

### 2.3. Eligibility Criteria

Studies were eligible if they met all of the following criteria: (1) the study was a randomized controlled trial (RCT); (2) participants had a confirmed diagnosis of COPD according to the Global Initiative for Chronic Obstructive Lung Disease (GOLD) criteria or other corresponding international or regional diagnostic standards, and the study sample had a mean age of ≥60 years; (3) the intervention group received a structured mind–body exercise intervention, including Tai Chi, Baduanjin, or Liuzijue; (4) the control group received usual treatment, routine care, health education, pulmonary rehabilitation, breathing training, another exercise intervention, or another comparator specified by the original study; and (5) the study reported at least one pulmonary function or exercise-capacity outcome that could be quantitatively synthesized, including forced expiratory volume in one second (FEV_1_), forced vital capacity (FVC), the FEV_1_/FVC ratio, or six-minute walk distance (6MWD).

Studies were excluded if they were non-randomized, if the independent effect of the mind–body exercise intervention could not be isolated, if COPD-specific data could not be separately extracted from mixed-disease samples, or if insufficient quantitative data were available for meta-analysis. For the overall analysis, eligible mind–body exercise interventions were treated as a broad intervention category, while exercise modality was examined separately as a moderator.

### 2.4. Data Extraction and Processing

Two reviewers independently extracted study characteristics, sample sizes, intervention and comparator characteristics, exercise modality, intervention duration, and outcome data for FEV_1_, FVC, FEV_1_/FVC, and 6MWD using a prespecified data extraction form. Disagreements were resolved through discussion, with consultation of a third reviewer when necessary.

Post-intervention means, standard deviations (SDs), and the corresponding analyzed sample sizes for the intervention and control groups were preferentially extracted.

When standard errors (SEs) were reported, they were converted to SDs using the formula SD = SE × √n. No additional model-based imputation of missing SDs was performed in the final statistical analyses. When intention-to-treat (ITT) estimates were reported, these were preferentially used. When only completer data were available, the analyzed sample sizes corresponding to the reported outcome statistics were used. Multiple reports originating from the same randomized trial were assigned a common study identifier to avoid double counting.

### 2.5. Risk-of-Bias Assessment

Risk of bias was assessed using the Cochrane Risk of Bias 2 (RoB 2) tool [17]. The following five domains were evaluated: (D1) bias arising from the randomization process; (D2) bias due to deviations from intended interventions; (D3) bias due to missing outcome data; (D4) bias in measurement of the outcome; and (D5) bias in selection of the reported result.

Each domain and the overall risk of bias were classified as low risk, some concerns, or high risk.

### 2.6. Effect Size Calculation

Hedges’ g, corrected for small-sample bias, was used as the primary standardized effect-size measure. Effect directions were harmonized such that g > 0 indicated an effect favoring the mind–body exercise group.

Because FEV_1_, FVC, and FEV_1_/FVC are measured on different scales, the overall pulmonary-function analysis was conducted using Hedges’ g, while outcome-specific analyses were additionally performed for each pulmonary-function measure.

To improve clinical interpretability, raw mean differences (MDs) were also calculated. FEV_1_ and FVC were expressed in liters (L), FEV_1_/FVC in percentage points, and 6MWD in meters (m).

### 2.7. Statistical Analysis

All statistical analyses were conducted in R version 4.6.1, primarily using the metafor package version 5.0–1 and the clubSandwich package version 0.7.0. All tests were two-sided, with *p* < 0.05 considered the conventional threshold for statistical significance. Where applicable, 95% confidence intervals (CIs) and 95% prediction intervals (PIs) were reported.

#### 2.7.1. Primary Meta-Analyses

Because individual studies could report multiple pulmonary-function outcomes, including FEV_1_, FVC, and FEV_1_/FVC, pulmonary function was analyzed using a three-level random-effects meta-analytic model. Level 1 represented sampling error, Level 2 represented residual heterogeneity among multiple effect sizes within the same study, and Level 3 represented between-study heterogeneity [14,18]. Variance components were estimated using restricted maximum likelihood (REML).

Because correlations among pulmonary-function outcomes within the same study were generally not reported, the sampling-error variance–covariance matrix for the primary analysis was constructed assuming a within-study correlation of ρ = 0.50. Statistical inference was further based on small-sample-adjusted cluster-robust variance estimation (CR2), with study as the clustering unit [19]. Within-study and between-study variance components, their corresponding I^2^ proportions, and 95% PIs were reported.

Because each study contributed only one 6MWD effect size, 6MWD was analyzed using a conventional two-level random-effects model with REML estimation and Knapp–Hartung inference. Both Hedges’ g and the raw MD in meters were reported.

For clinical interpretation of the original measurement scales, FEV_1_, FVC, and FEV_1_/FVC were additionally synthesized separately using outcome-specific two-level random-effects models with REML estimation and Knapp–Hartung inference. Raw MDs were not pooled across pulmonary-function outcomes measured on different scales.

#### 2.7.2. Subgroup Analyses and Meta-Regression

Exploratory subgroup analyses were conducted according to pulmonary-function outcome, exercise modality, and intervention duration. Exercise modality was categorized as Baduanjin, Liuzijue, or Tai Chi. Intervention duration was categorized as ≤3 months, >3 to <12 months, or ≥12 months.

For pulmonary-function outcomes, subgroup differences were evaluated using joint Wald tests within the three-level model with CR2 inference. For 6MWD, overall moderator effects were evaluated using two-level random-effects meta-regression. Comparisons among exercise modalities or intervention-duration categories were based on formal omnibus moderator tests rather than on the statistical significance or magnitude of individual subgroup estimates.

For the five categorical moderator tests, multiplicity was controlled using the Holm procedure. Benjamini–Hochberg false discovery rate (FDR)-adjusted q values were also reported.

Intervention duration was further modeled as a continuous variable centered at 6 months. Separate linear and quadratic meta-regression models were fitted. Pulmonary-function meta-regressions used the three-level model with CR2 inference, whereas 6MWD meta-regressions used two-level random-effects models with Knapp–Hartung inference. Regression coefficients were estimated using REML. Maximum likelihood (ML) models were used only for comparisons of the Akaike information criterion (AIC) and the Bayesian information criterion (BIC) between linear and quadratic model specifications. Non-intercept duration terms were also subjected to multiplicity correction.

#### 2.7.3. Sensitivity Analyses

For pulmonary function, sensitivity analyses were conducted assuming within-study correlations of ρ = 0, 0.20, 0.50, and 0.80 to evaluate the robustness of the findings to the assumed correlation structure. REML and ML estimates were also compared, and leave-one-study-out analyses were performed.

Potentially influential observations were identified using an absolute studentized residual threshold of ∣studentized residual∣ > 2.58, after which the corresponding studies were excluded and the models were refitted.

For 6MWD, REML and ML estimates were similarly compared. Leave-one-study-out analyses were performed for the raw-MD model, together with sensitivity analyses based on the same ∣studentized residual∣ > 2.58 criterion.

#### 2.7.4. Small-Study Effects

Funnel plots were constructed separately for FEV_1_, FVC, FEV_1_/FVC, and 6MWD. Funnel-plot asymmetry was explored using Egger’s regression test and Begg’s rank-correlation test, and trim-and-fill analyses were additionally performed.

Because funnel-plot asymmetry may arise from sources other than publication bias, including heterogeneity and differences in study design, these analyses were interpreted as exploratory diagnostics of small-study effects. A statistically significant result from any single test was not considered definitive evidence of publication bias.

## 3. Results

### 3.1. Study Selection and Characteristics

**Figure 1:**
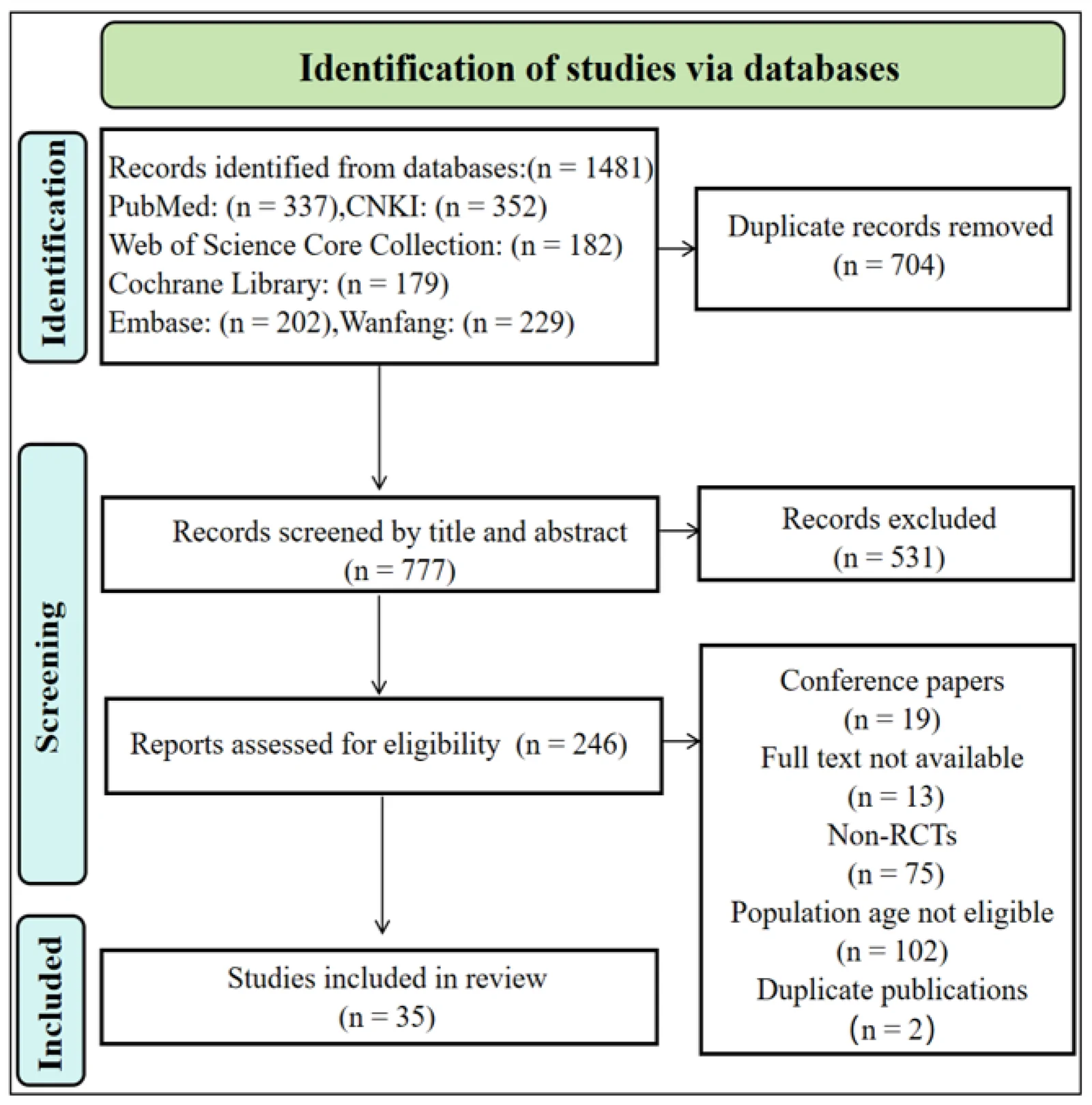
PRISMA flow diagram of the study-selection process.

A total of 35 independent studies involving 2955 patients with COPD were included, of whom 1484 were allocated to the intervention groups and 1471 to the control groups. Overall, 85 effect sizes were included. The pulmonary-function analysis comprised 65 effect sizes from 28 studies, whereas the six-minute walk distance (6MWD) analysis comprised 20 effect sizes from 20 studies. The pulmonary-function effect sizes included 26 for FEV_1_, 16 for FVC, and 23 for FEV_1_/FVC. The included studies evaluated three types of mind–body exercise—Liuzijue, Baduanjin, and Tai Chi—with intervention durations ranging from 1 to 12 months. The study-selection process is shown in Figure 1, and the characteristics of the included studies are summarized in Table 1.

### 3.2. Risk of Bias

**Figure 2:**
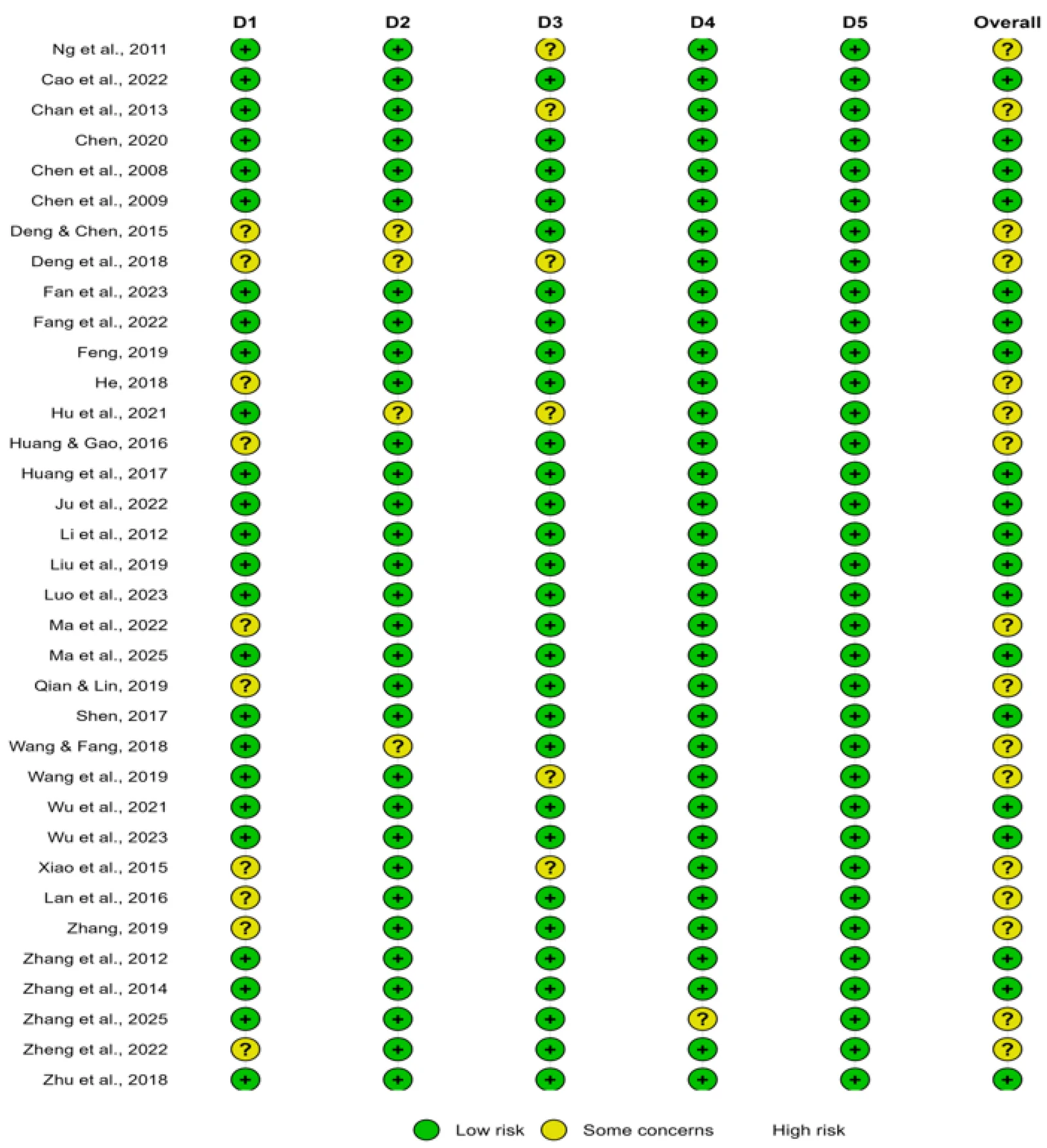
Risk-of-bias judgments for the 35 included studies [20,21,22,23,24,25,26,27,28,29,30,31,32,33,34,35,36,37,38,39,40,41,42,43,44,45,46,47,48,49,50,51,52,53,54] using the Cochrane RoB 2 toolD1, bias arising from the randomization process; D2, bias due to deviations from intended interventions; D3, bias due to missing outcome data; D4, bias in measurement of the outcome; D5, bias in selection of the reported result.

Among the 35 included studies, 19 (54.3%) were judged to have an overall low risk of bias, whereas 16 (45.7%) were judged to have some concerns; none were rated as having a high risk of bias. At the domain level, the randomization process (D1) was the main source of uncertainty, with 25 studies (71.4%) rated as low risk and 10 (28.6%) were rated as having some concerns. For deviations from intended interventions (D2), 31 studies (88.6%) were rated as low risk and four (11.4%) as having some concerns. For missing outcome data (D3), 29 studies (82.9%) were rated as low risk and 6 (17.1%) were rated as having some concerns. Only one study was judged to have some concerns regarding the measurement of the outcome (D4), whereas all 35 studies were rated as low risk for selection of the reported result (D5). Detailed judgments are presented in Figure 2.

### 3.3. Overall Effects of Mind–Body Exercise on Pulmonary Function

**Figure 3:**
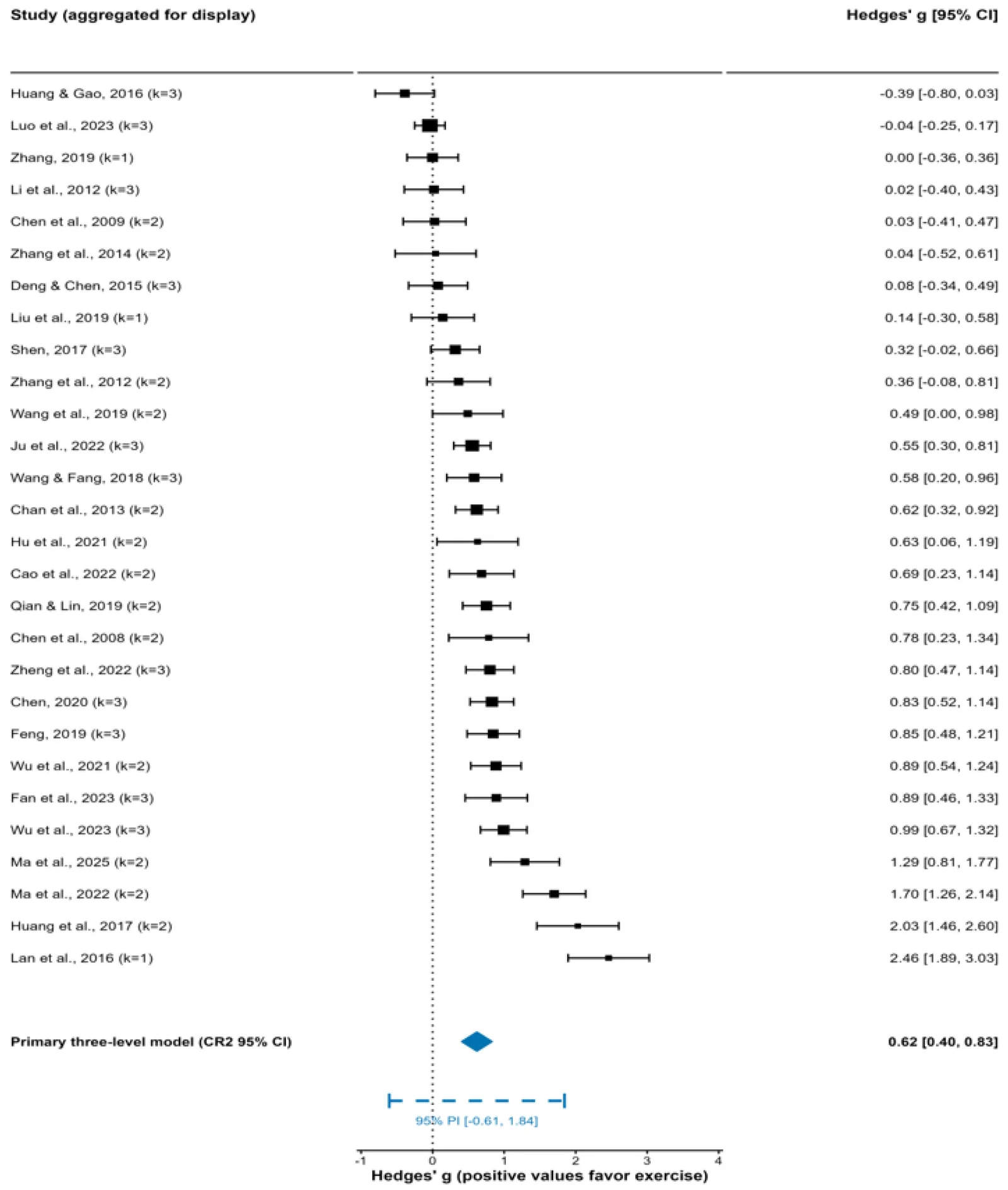
Study-level display of pulmonary-function effects and the primary three-level meta-analysis [21,22,23,24,25,26,28,30,32,33,34,35,36,37,38,39,40,41,42,43,44,45,46,48,49,50,51,53]. For studies reporting multiple pulmonary-function outcomes, Hedges’ g values for FEV_1_, FVC, and/or FEV_1_/FVC were aggregated with equal weights at the study level solely for graphical presentation. These study-level aggregated values were not used to refit the primary model. The pooled diamond, 95% CI, and 95% PI were derived from the primary three-level model including all 65 pulmonary-function effect sizes.

Twenty-eight studies contributed 65 effect sizes for pulmonary function. For visual clarity, Figure 3 presents study-level aggregated effects, whereas the primary three-level model was fitted using all 65 original effect sizes. The complete effect-level forest plot is provided in Supplementary Figure S1.

Using a three-level random-effects model assuming a within-study correlation of ρ = 0.50, together with study-clustered CR2 robust inference, mind–body exercise showed a significant positive overall effect on pulmonary function (Hedges’ g = 0.62, 95% CI 0.40–0.83, *p* < 0.001). On the original clinical scales, FEV_1_ increased by a mean of 0.22 L (95% CI 0.15–0.29; 95% PI −0.10–0.54), FVC by 0.28 L (95% CI 0.15–0.40; 95% PI −0.15–0.71), and FEV_1_/FVC by 3.35 percentage points (95% CI 1.66–5.05; 95% PI −3.73–10.43). Although the average effects for all three outcomes were positive, each prediction interval included the null value. For overall pulmonary function, the 95% PI ranged from −0.61 to 1.84 and also crossed the null, indicating substantial uncertainty in the true effect that might be observed in a future comparable study.

Heterogeneity in the pulmonary-function model was substantial, with a total I^2^ of 91.0%. Under the primary assumption of ρ = 0.50, approximately 49.9% of the total variability was attributable to between-study heterogeneity and 41.1% to within-study heterogeneity among effect sizes. Residual heterogeneity was also statistically significant.

### 3.4. Overall Effects of Mind–Body Exercise on 6MWD

**Figure 4:**
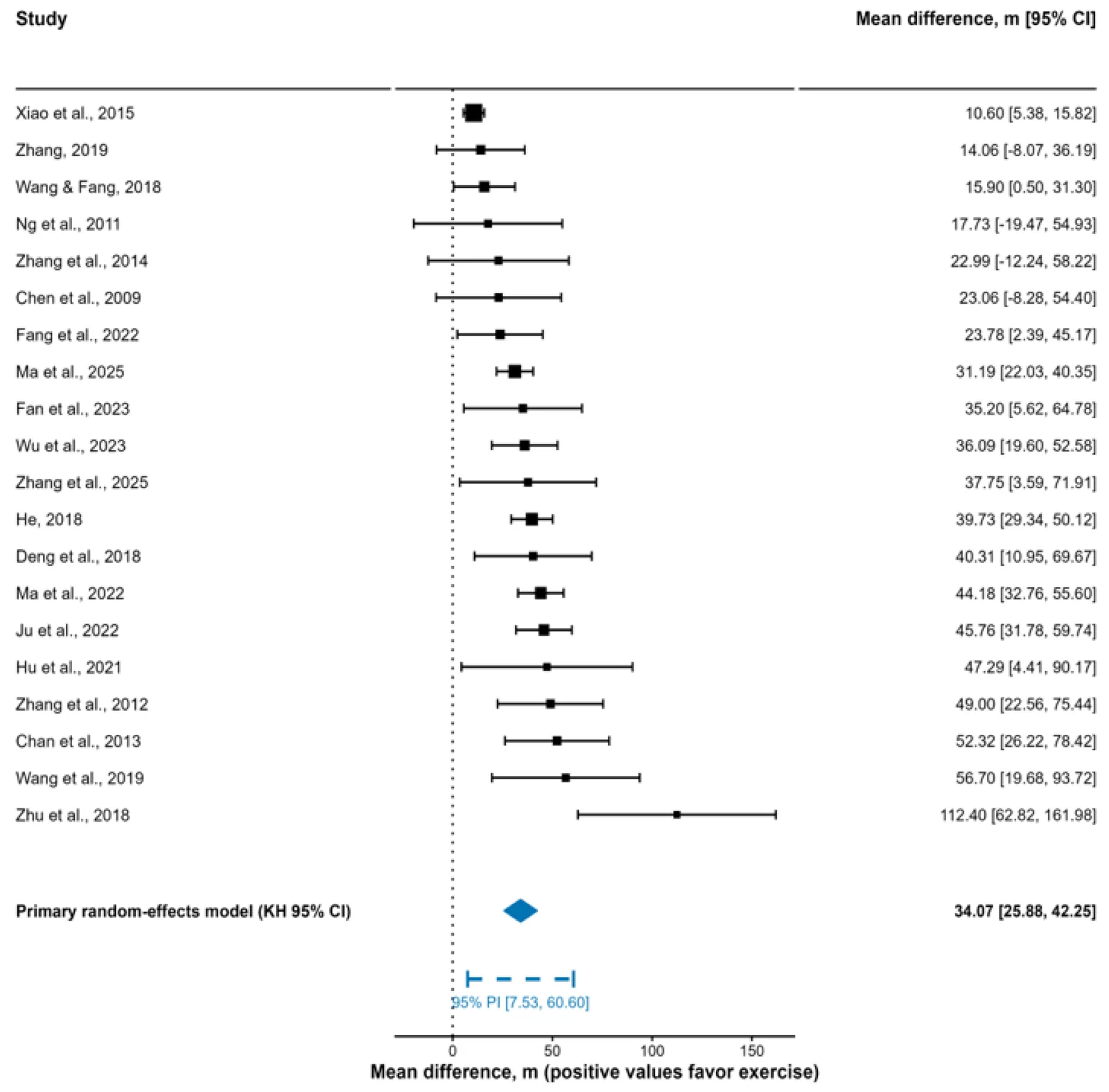
Individual-study and pooled raw mean differences in 6MWD [20,22,25,27,28,29,31,32,35,39,40,43,44,46,47,49,50,51,52,54]. Squares and horizontal lines represent individual-study estimates and their 95% CIs, respectively. The diamond represents the pooled random-effects estimate with Knapp–Hartung adjustment, and the dashed line indicates the model-based 95% prediction interval.

Twenty studies were included in the 6MWD analysis (Figure 4). The two-level random-effects model with Knapp–Hartung inference showed that mind–body exercise significantly improved 6MWD, with a standardized effect of Hedges’ g = 0.78 (95% CI 0.57–0.99, *p* < 0.001). The corresponding 95% PI ranged from −0.02 to 1.57.

On the more clinically interpretable original scale, the mind–body exercise groups achieved a mean improvement in 6MWD of 34.07 m relative to the control groups (95% CI 25.88–42.25 m), with a 95% PI of 7.53–60.60 m and an I^2^ of 68.7%. The principal pooled estimates, 95% CIs, 95% PIs, and heterogeneity statistics for pulmonary function and 6MWD are summarized in Table 2.

### 3.5. Exploratory Subgroup and Moderator Analyses

#### 3.5.1. Pulmonary Function

When stratified by pulmonary-function outcome, the pooled effects for FEV_1_, FVC, and FEV_1_/FVC were g = 0.71 (95% CI 0.40–1.02), 0.52 (95% CI 0.28–0.76), and 0.53 (95% CI 0.25–0.81), respectively. The overall difference among the three pulmonary-function outcomes was not statistically significant (omnibus *p* = 0.187; Holm-adjusted *p* = 0.560).

When stratified by exercise modality, the point estimates for Baduanjin, Liuzijue, and Tai Chi were g = 0.80 (95% CI 0.26–1.34), 0.68 (95% CI 0.38–0.98), and 0.24 (95% CI −0.10–0.57), respectively. The unadjusted omnibus test for exercise modality yielded *p* = 0.048; however, this association was no longer statistically significant after Holm correction (*p* = 0.240).

When stratified by intervention duration, the pooled effects for interventions lasting ≤3 months, >3 to <12 months, and ≥12 months were g = 0.68, 0.68, and 0.07, respectively. The unadjusted omnibus test yielded *p* = 0.048, but the Holm-adjusted *p* value was 0.240.

**Figure 5:**
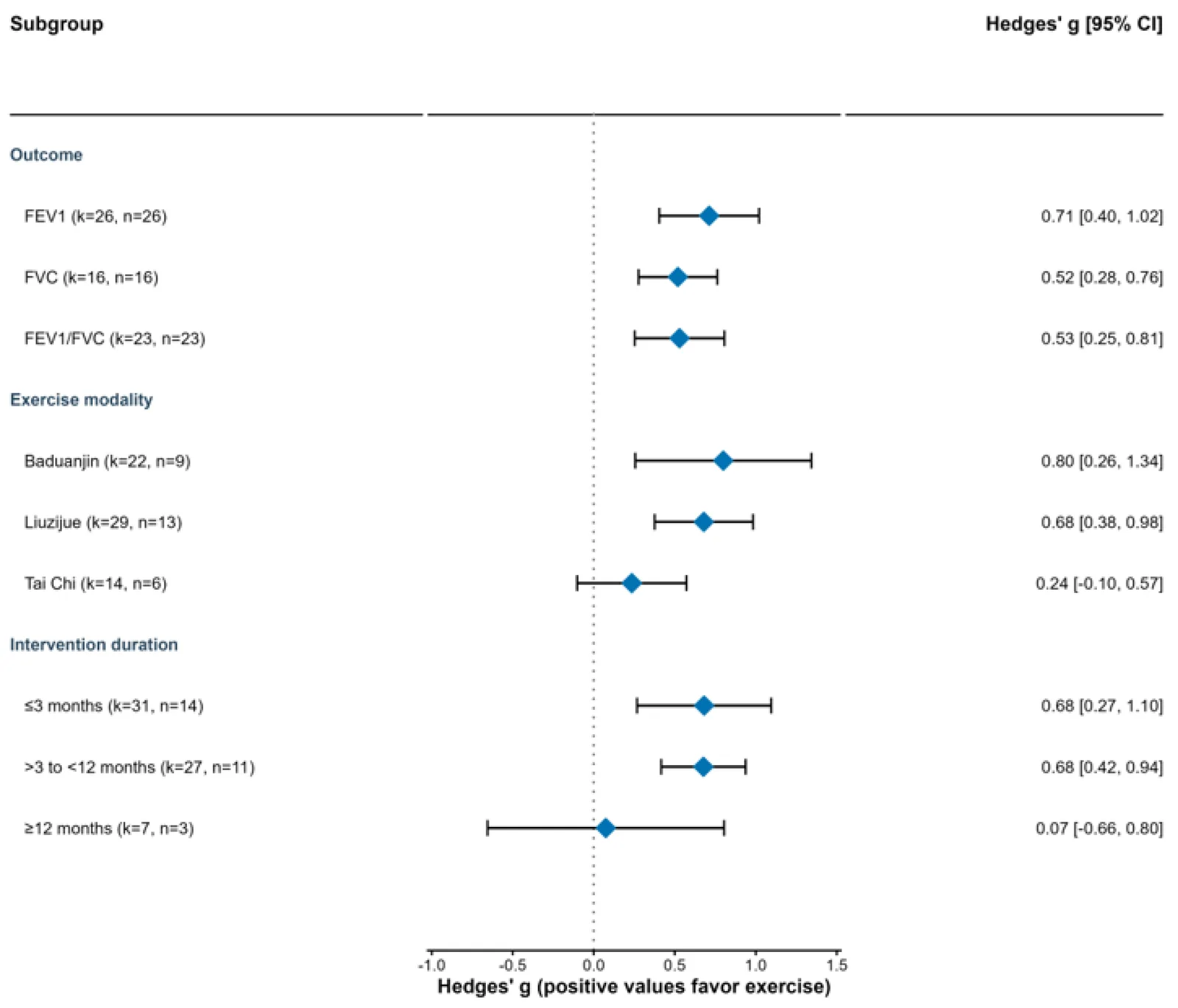
Exploratory subgroup analyses for pulmonary function. k denotes the number of effect sizes and n the number of independent studies. Comparisons among subgroups were based on formal omnibus moderator tests and Holm-adjusted results rather than on the magnitude or statistical significance of individual subgroup estimates.

Accordingly, after correction for multiple comparisons, there was no robust evidence of differences among pulmonary-function outcomes, exercise modalities, or intervention-duration subgroups (Figure 5). Complete subgroup estimates, omnibus moderator tests, and multiplicity-adjusted results are provided in Supplementary Table S2.

#### 3.5.2. 6MWD

**Figure 6:**
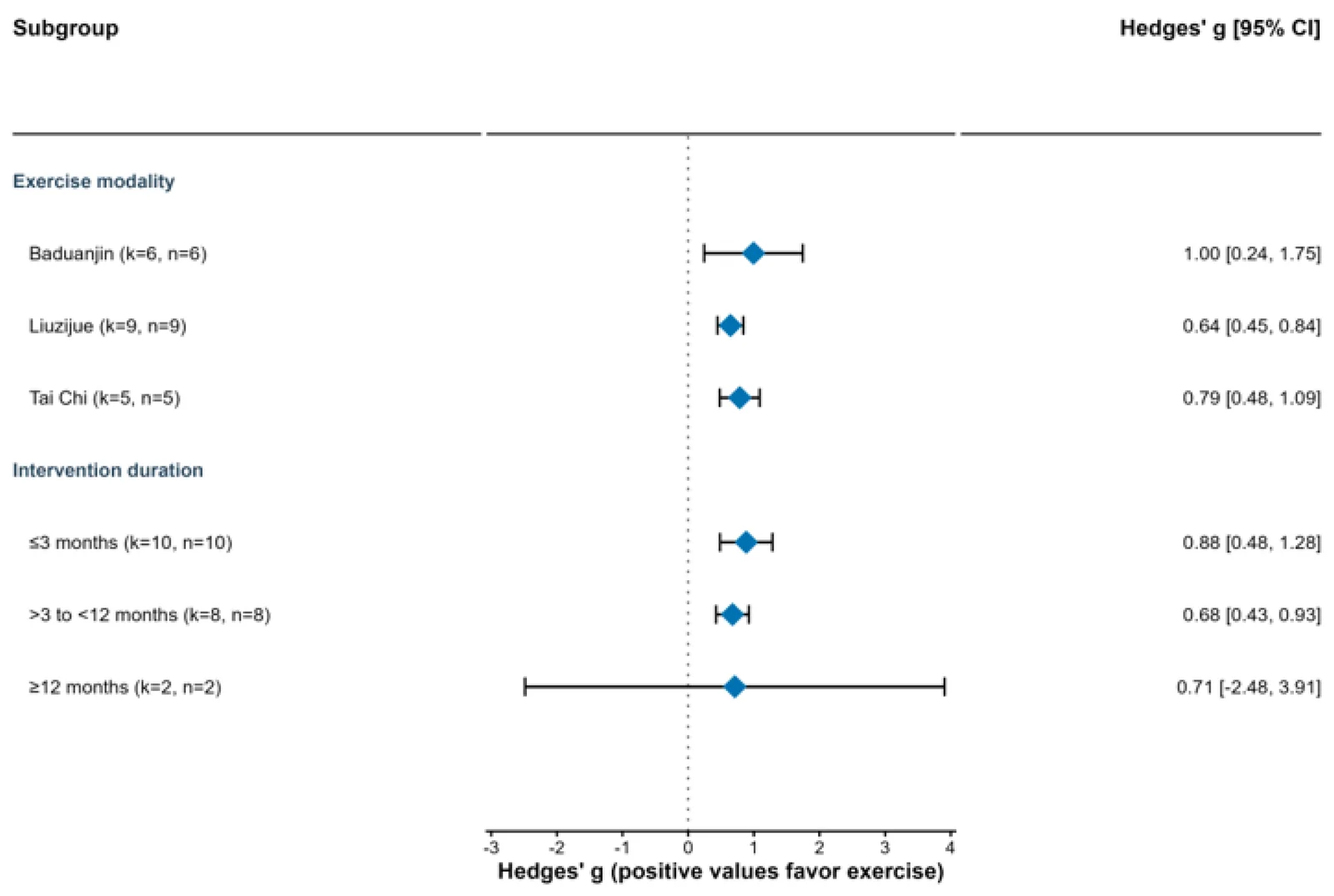
Exploratory subgroup analyses for 6MWD. Between-subgroup differences according to exercise modality and intervention duration were not supported by the formal omnibus moderator tests.

Exploratory subgroup analyses of 6MWD according to exercise modality and intervention duration are shown in Figure 6. By exercise modality, the standardized effects for Baduanjin, Liuzijue, and Tai Chi were g = 1.00 (95% CI 0.24–1.75), 0.64 (95% CI 0.45–0.84), and 0.79 (95% CI 0.48–1.09), respectively. However, the overall moderator effect was not statistically significant (*p* = 0.372; Holm-adjusted *p* = 0.744).

By intervention duration, the pooled effects for interventions lasting ≤3 months, >3 to <12 months, and ≥12 months were g = 0.88 (95% CI 0.48–1.28), 0.68 (95% CI 0.43–0.93), and 0.71 (95% CI −2.48–3.91), respectively. The overall difference was likewise not statistically significant (*p* = 0.637; Holm-adjusted *p* = 0.744). Notably, the ≥12-month subgroup included only two studies and was therefore estimated with considerable imprecision.

### 3.6. Continuous Meta-Regression of Intervention Duration

Continuous meta-regression provided no robust evidence of either linear or quadratic associations between intervention duration and the effects on pulmonary function or 6MWD. For pulmonary function, the *p* values for the linear and quadratic duration terms were 0.142 and 0.199, respectively; for 6MWD, the corresponding *p* values were 0.443 and 0.196. After correction for multiple comparisons, none of the non-intercept duration terms reached statistical significance.

**Figure 7:**
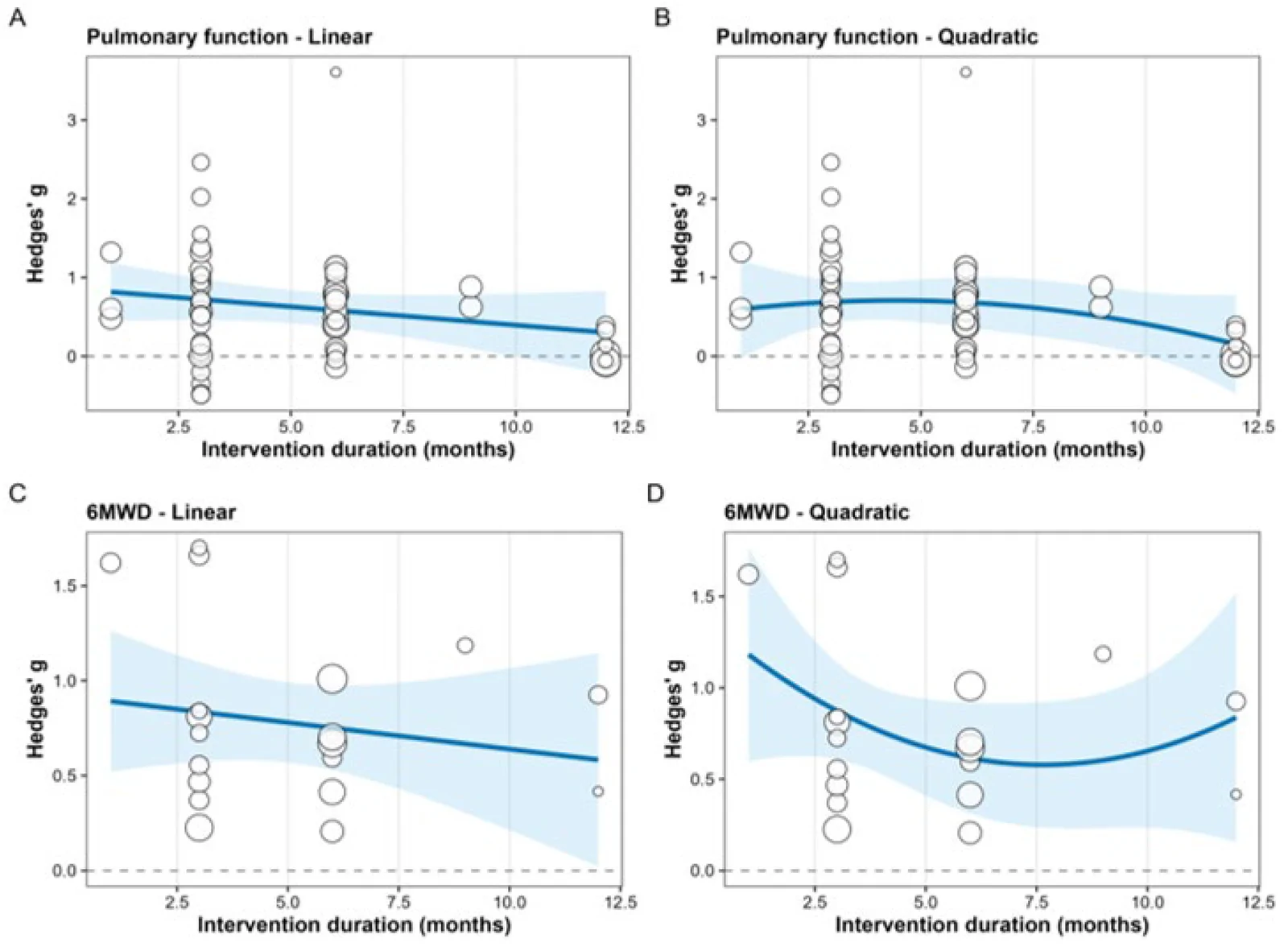
Exploratory linear and quadratic meta-regression analyses of intervention duration. (**A**) Linear meta-regression for pulmonary function; (**B**) quadratic meta-regression for pulmonary function; (**C**) linear meta-regression for 6MWD; (**D**) quadratic meta-regression for 6MWD. Solid lines represent fitted values, and shaded areas represent 95% CIs. The fitted curves should not be interpreted as evidence of an established optimal intervention duration.

Model-fit indices likewise provided no clear evidence supporting a stable quadratic dose–response pattern (Figure 7). Complete regression coefficients, 95% CIs, multiplicity-adjusted results, and AIC/BIC statistics are provided in Supplementary Table S3.

### 3.7. Small-Study Effects

The findings of the small-study-effect diagnostics were not fully consistent across outcomes. For FEV_1_, Egger’s regression test indicated funnel-plot asymmetry (*p* = 0.002), whereas Begg’s test did not reach conventional statistical significance (*p* = 0.057), and the trim-and-fill procedure did not impute any missing studies. For FVC, Egger’s and Begg’s tests yielded *p* = 0.796 and *p* = 0.965, respectively, whereas trim-and-fill imputed three potentially missing studies. For FEV_1_/FVC, the corresponding *p* values were 0.899 and 0.601, and trim-and-fill imputed six potentially missing studies. For 6MWD, Egger’s and Begg’s tests yielded *p* = 0.285 and *p* = 0.260, respectively, and no studies were imputed by trim-and-fill.

Given the substantial heterogeneity and inconsistency among diagnostic methods, these findings were interpreted as exploratory indicators of small-study effects rather than as definitive evidence for or against publication bias. Funnel plots and trim-and-fill analyses are shown in Supplementary Figure S2, and the complete results of Egger’s test, Begg’s test, and trim-and-fill analyses are provided in Supplementary Table S4.

### 3.8. Sensitivity Analyses

The primary pulmonary-function effect was robust to alternative assumptions regarding the within-study correlation. At ρ = 0, 0.20, 0.50, and 0.80, the pooled effect remained approximately g = 0.62, with all 95% CIs excluding the null. REML and ML estimates were also highly consistent. After excluding studies containing observations with ∣studentized residual∣ > 2.58, the pooled pulmonary-function effect remained positive at g = 0.53 (95% CI 0.35–0.72). Corresponding results are presented in Supplementary Figure S3.

The 6MWD results were similarly robust. Standardized effect estimates from the REML and ML models were both approximately g = 0.78. For the raw-MD model, leave-one-study-out pooled estimates ranged from 32.49 to 36.09 m. After excluding studies containing observations exceeding the prespecified studentized-residual threshold, the pooled MD was 35.07 m (95% CI 29.22–40.93). Sensitivity analyses for 6MWD on the original scale are shown in Supplementary Figure S4, and the complete sensitivity-analysis results are provided in Supplementary Table S5.

Overall, the principal findings were not driven by the assumed correlation structure, estimation method, or any single study.

## 4. Discussion

### 4.1. Principal Findings and Clinical Interpretation

This meta-analysis included 35 independent studies contributing 85 effect sizes and systematically evaluated the effects of mind–body exercise on pulmonary function and exercise capacity in patients with COPD from samples with a mean age of ≥60 years. Overall, mind–body exercise improved both pulmonary function and six-minute walk distance (6MWD). On the original clinical scales, the mean improvements were approximately 0.22 L for FEV_1_, 0.28 L for FVC, 3.35 percentage points for FEV_1_/FVC, and 34.07 m for 6MWD. Taken together, these findings support an overall rehabilitative benefit of mind–body exercise. However, the prediction intervals indicate substantial context-dependent variability: the 95% PI for overall pulmonary function ranged from −0.61 to 1.84, whereas that for the standardized 6MWD effect ranged from −0.02 to 1.57. Thus, the findings are more appropriately interpreted as evidence of benefit on average, rather than as evidence that consistent benefits can be expected across all patients and implementation settings.

The raw-scale 6MWD result has particular clinical relevance. Previous studies have suggested a minimal clinically important difference (MCID) of approximately 25–35 m for 6MWD in patients with COPD [55,56]. The mean between-group difference of 34.07 m observed in the present meta-analysis falls within this range, suggesting that the average benefit may approach a clinically perceptible magnitude. However, MCID estimates are primarily derived from within-patient changes over time, whereas the present meta-analysis estimated an average between-group difference across trials; these quantities are not directly equivalent. Moreover, the 95% PI for the raw 6MWD MD ranged from 7.53 to 60.60 m, with its lower bound well below commonly used MCID thresholds. Therefore, an average effect that reaches or approaches the MCID should not be interpreted as indicating that all future programs will produce clinically important improvements.

The pulmonary-function findings require more cautious interpretation. FEV_1_, FVC, and FEV_1_/FVC reflect different aspects of respiratory physiology, and persistent airflow limitation in COPD is associated with pathological changes including small-airway remodeling, parenchymal destruction, and loss of elastic recoil [2]. Mind–body exercise may influence some pulmonary-function measures through breathing control, respiratory muscle function, thoracic mobility, and respiratory–motor coordination; however, the present findings do not imply that such exercise can reverse the persistent airflow limitation or structural lung abnormalities associated with COPD. Changes in pulmonary function should therefore be interpreted alongside dyspnea, quality of life, physical activity, and 6MWD rather than as a stand-alone indicator of therapeutic efficacy.

From a clinical perspective, mind–body exercise should also not be regarded as a replacement for standard pulmonary rehabilitation. Contemporary pulmonary rehabilitation is centered on individualized aerobic and resistance exercise training, education, and behavioral support [6,7]. Tai Chi, Baduanjin, and Liuzijue are low-impact modalities that require relatively little equipment, can be practiced in community or home settings, and integrate breathing regulation with movement. However, the present meta-analysis did not test the equivalence or non-inferiority of these modalities relative to a complete pulmonary-rehabilitation program. The available evidence therefore more appropriately supports their use as adjunctive or optional exercise modalities within comprehensive pulmonary rehabilitation.

The overall direction of benefit observed in the present study is broadly consistent with previous systematic reviews and meta-analyses, which have also suggested that mind–body or traditional Chinese exercises may improve pulmonary function and exercise capacity in patients with stable COPD [9,12]. The present study extends this literature by focusing on samples with a mean age of ≥60 years and by retaining multiple correlated pulmonary-function outcomes within a three-level meta-analytic framework. Notably, a previous network meta-analysis suggested potential rankings among different traditional exercise modalities [12], whereas the present study found no robust differences among exercise modalities after formal moderator testing and adjustment for multiple comparisons. This discrepancy may reflect differences in the populations included, study scope, effect-size handling, and comparative framework. Accordingly, the more cautious interpretation is that different traditional exercise modalities may have rehabilitative value, but current evidence remains insufficient to establish a clinically meaningful efficacy hierarchy.

### 4.2. Heterogeneity and Outcome-Specific Interpretation

Substantial heterogeneity was observed for pulmonary function, with a total I^2^ of 91.0%. Under the primary assumption of ρ = 0.50, approximately 49.9% of the total variability was attributable to between-study heterogeneity and 41.1% to heterogeneity among effect sizes within studies. This pattern suggests that variation in treatment effects may arise both from differential responses across FEV_1_, FVC, and FEV_1_/FVC within the same study and from between-study differences in participant characteristics, exercise protocols, comparator conditions, and measurement procedures.

Because most primary studies did not report correlations among different pulmonary-function outcomes, the within-study sampling covariance structure had to be constructed using a prespecified value of ρ. The principal purpose of the three-level model was therefore to account appropriately for the statistical dependence among correlated effect sizes; the estimated proportions of variance at different levels should not be interpreted as established physiological mechanisms [13,14]. Importantly, the overall pulmonary-function effect remained approximately g = 0.62 when ρ was varied from 0 to 0.80, indicating that the primary conclusion was robust to alternative assumptions regarding within-study correlation.

When stratified by pulmonary-function outcome, the pooled effects for FEV_1_, FVC, and FEV_1_/FVC were 0.71, 0.52, and 0.53, respectively, but the overall difference was not statistically significant. Thus, the current evidence does not establish that any one pulmonary-function measure is more responsive to mind–body exercise, and the relative magnitude of standardized effect sizes should not be used to infer differential sensitivity.

For 6MWD, each of the 20 studies contributed a single effect size, allowing the use of a conventional two-level model. Heterogeneity remained substantial, with I^2^ values of 71.0% for the standardized model and 68.7% for the raw-MD model. This indicates that between-study differences in disease severity, training dose, adherence, testing procedures, and comparator conditions may remain important. It also reinforces the need to interpret pooled mean effects together with prediction intervals. In the presence of substantial heterogeneity, prediction intervals provide more direct information about the uncertainty surrounding effects that might be expected in future settings than the pooled mean effect or I^2^ alone [16].

### 4.3. Exercise Modality

When stratified by exercise modality, the point estimates for pulmonary function were g = 0.80 for Baduanjin, 0.68 for Liuzijue, and 0.24 for Tai Chi. Although the unadjusted omnibus test yielded *p* = 0.048, the difference was no longer statistically significant after Holm correction. For 6MWD, the corresponding effect estimates were 1.00, 0.64, and 0.79, and the overall moderator test for exercise modality was also not statistically significant. Thus, the current evidence does not support concluding that any one modality is superior to the others.

This distinction is important because these subgroup estimates were derived primarily from indirect between-study comparisons rather than from head-to-head comparisons within the same randomized trial. Between-study subgroup comparisons can be confounded by differences in patient characteristics, exercise dose, comparator conditions, supervision, and other study-level characteristics. Accordingly, differences among subgroups should be evaluated using formal moderator tests rather than by ranking point estimates or comparing the statistical significance of individual subgroup effects [16].

Nevertheless, the distinct training characteristics of these modalities may provide a basis for mechanistic hypotheses. Liuzijue emphasizes vocalization, slow expiration, and breathing control. A recent surface-electromyography study in healthy, experienced Liuzijue practitioners identified characteristic patterns of respiratory-muscle activation during practice; however, whether these findings generalize to patients with COPD remains to be established [57]. Baduanjin incorporates upper-limb and trunk movements, knee flexion, and weight shifting, and long-term practice may influence lower-limb strength, balance, and gait control [58]. Tai Chi places greater emphasis on continuous weight shifting, multijoint coordination, and balance control [8,11]. These characteristics suggest that different forms of mind–body exercise may exert their effects through differing relative contributions of respiratory training, peripheral muscle function, and neuromuscular coordination.

### 4.4. Intervention Duration

For pulmonary function, the pooled effects for interventions lasting ≤3 months, >3 to <12 months, and ≥12 months were g = 0.68, 0.68, and 0.07, respectively. Although the unadjusted omnibus test yielded *p* = 0.048, the difference was no longer statistically significant after Holm correction.

The number of long-duration studies was limited, and exercise modality and intervention duration were not independently distributed across the evidence base. Consequently, the lower point estimate observed in the long-duration subgroup may reflect differences in modality composition, participant characteristics, and study design rather than a true attenuation of efficacy over time.

Continuous meta-regression provided no significant evidence of either linear or quadratic associations between intervention duration and pulmonary function (linear *p* = 0.142; quadratic *p* = 0.199) or 6MWD (linear *p* = 0.443; quadratic *p* = 0.196). These conclusions were unchanged after correction for multiple comparisons. The fitted curves should therefore be regarded as exploratory descriptions rather than as evidence of a defined dose–response pattern.

Similarly, no statistically significant duration-related differences were identified for 6MWD, and the ≥12-month subgroup contained only two studies, resulting in a very wide 95% CI. The most appropriate conclusion is therefore that current evidence is insufficient to identify an optimal intervention duration. Future trials should evaluate duration effects directly within the same study using standardized exercise modalities and doses and multiple prespecified follow-up assessments, rather than relying on indirect comparisons of duration categories across different studies.

### 4.5. Small-Study Effects, Risk of Bias, and Robustness

The different diagnostic approaches to small-study effects did not yield fully consistent findings. For FEV_1_, Egger’s test suggested funnel-plot asymmetry, whereas Begg’s test was not statistically significant and the trim-and-fill procedure did not impute any missing studies. Egger’s tests for FVC, FEV_1_/FVC, and 6MWD did not indicate clear asymmetry, whereas trim-and-fill nevertheless imputed potentially missing studies for some pulmonary-function outcomes. These findings are therefore more appropriately regarded as exploratory signals of small-study effects rather than as evidence establishing either the presence or absence of publication bias. Funnel-plot asymmetry may also arise from genuine heterogeneity, differences in study design, or mathematical relationships between effect estimates and their standard errors [16]. Moreover, because the present study primarily used standardized effect sizes based on Hedges’ g, the potential mathematical association between standardized mean differences and their standard errors warrants particular caution when interpreting Egger’s test [16].

The main findings were relatively stable across sensitivity analyses. The pooled pulmonary-function effect remained approximately g = 0.62 across different values of ρ and under REML and ML estimation. After excluding studies containing observations with ∣studentized residual∣ > 2.58, the effect remained positive (g = 0.53, 95% CI 0.35–0.72). For the raw 6MWD analysis, leave-one-study-out pooled MDs ranged from 32.49 to 36.09 m, and the sensitivity estimate after exclusion based on the prespecified residual threshold was 35.07 m (95% CI 29.22–40.93). These findings indicate that the main conclusions were not driven by any single study or specific statistical assumption. Nevertheless, statistical robustness does not eliminate uncertainty arising from the methodological quality or external validity of the underlying studies.

The RoB 2 assessment showed that 19 of the 35 studies (54.3%) were judged to have an overall low risk of bias, whereas 16 (45.7%) had some concerns; none were judged to have a high risk of bias. The main concerns involved the randomization process, missing outcome data, and deviations from intended interventions. Thus, although the evidence base was not characterized by a high overall risk of bias, the magnitude of the pooled effects and subgroup differences should still be interpreted cautiously.

### 4.6. Strengths and Limitations

A major strength of this study is that the statistical models were selected according to the actual dependence structure of the data. For pulmonary function, multiple outcomes from the same study were retained and analyzed using a three-level random-effects model with study-clustered CR2 robust inference, whereas 6MWD was analyzed using a two-level random-effects model with Knapp–Hartung inference. In addition, the study reported 95% prediction intervals and effects on clinically interpretable original scales, applied multiplicity correction to subgroup and meta-regression analyses, and evaluated robustness across alternative values of ρ, estimation methods, residual-based exclusions, and leave-one-study-out analyses.

Several limitations should also be acknowledged. First, the available evidence was derived predominantly from Chinese populations or populations with a Chinese cultural background. Cultural familiarity with traditional exercises, instructional approaches, and participant expectations may influence adherence and treatment response, thereby limiting generalizability to other ethnic populations and healthcare systems. Second, substantial between-study variation existed in disease severity, pharmacological treatment, exercise dose, supervision, and comparator conditions, whereas available information was insufficient to support more detailed moderator analyses. Third, exercise modality and intervention duration were not orthogonally distributed, creating potential confounding between modality and duration effects. Fourth, comparisons among exercise modalities were primarily between studies rather than within head-to-head randomized trials; subgroup effect sizes therefore cannot be used to establish a hierarchy of efficacy.

Fifth, some subgroups included relatively few studies, particularly those involving Tai Chi and long-duration interventions. Wide confidence intervals in these subgroups neither demonstrate a lack of effect nor establish equivalence. Sixth, pulmonary-function heterogeneity was substantial, and the partitioning of variance across model levels depended on the prespecified within-study correlation. Seventh, reporting of adherence, actual training dose, movement quality, adverse events, and long-term maintenance was limited. Finally, the FEV_1_ analysis showed a signal of small-study effects; therefore, selective publication or disproportionate contributions from small positive studies cannot be completely excluded.

### 4.7. Clinical Implications and Future Research

Following appropriate safety screening and individualized rehabilitation assessment, Tai Chi, Baduanjin, and Liuzijue may be considered as adjunctive or optional exercise modalities within comprehensive pulmonary rehabilitation rather than as replacements for standard pulmonary rehabilitation [6,7]. Their relatively low equipment requirements may make them particularly feasible for some patients who face transportation or equipment barriers, prefer low-impact exercise, or require home- or community-based training [7,8]. Selection of a specific modality should be guided by individual needs for breathing control, balance and lower-limb function, ability to learn movement sequences, patient preferences, and access to qualified instruction rather than by indirect subgroup effect estimates.

Future research should first prioritize adequately powered multicenter RCTs outside China to evaluate acceptability, adherence, and physiological responses across different cultural and healthcare contexts. Second, head-to-head trials comparing Liuzijue, Baduanjin, and Tai Chi are needed. Direct comparisons with standard aerobic exercise, progressive resistance training, inspiratory muscle training, and other structured mind–body or breathing-based approaches, such as Yoga, Pilates, or structured breathing training, would also help clarify their practical value as adjunctive components of pulmonary rehabilitation or as more accessible exercise options.

Future trials should consistently report FEV_1_, FVC, FEV_1_/FVC, 6MWD, dyspnea, quality of life, adverse events, and adherence, and should incorporate MCID-based interpretation or responder analyses to improve clinical interpretability. Exercise dose should be documented in terms of actual training frequency, session duration, intensity, completed volume, and progression strategies. Multiple prespecified follow-up assessments would allow dose–response relationships to be examined directly.

Mechanistic studies should concurrently assess respiratory muscle function, diaphragmatic activity, peripheral muscle function, physical activity, autonomic indices, inflammatory biomarkers, and dyspnea perception and should use prespecified mediation analyses to test potential pathways. Only after consistent findings are demonstrated in high-quality direct-comparison studies across different regions would there be a sufficient evidence base to recommend a specific exercise modality or fixed training duration.

## 5. Conclusions

This meta-analysis indicates that, on average, mind–body exercise may improve pulmonary function and functional exercise capacity in older adults with COPD, suggesting potential clinical relevance. However, the substantial heterogeneity and prediction intervals indicate that the magnitude of benefit may vary across patient populations and implementation settings. Differences among exercise modalities and intervention durations were not supported by robust statistical evidence after correction for multiple comparisons; therefore, the current evidence is insufficient to identify an optimal exercise modality or intervention duration.

Tai Chi, Baduanjin, and Liuzijue may be considered as adjunctive or optional exercise modalities within comprehensive pulmonary rehabilitation, with the choice of modality guided by patient characteristics, preferences, and feasibility of implementation. Future cross-cultural, multicenter, and head-to-head randomized controlled trials are needed to clarify the comparative effectiveness of different exercise modalities, optimal training dose, and long-term clinical value.

## Figures and Tables

**Table 1:** Characteristics of the included studies.

Study	Publication Language	COPD Duration/Severity	Sample Size (Exercise/Control)	Exercise Intervention	Control Intervention	Duration (Months)	Outcomes
Ng et al., 2011 [20]	English	FEV_1_ predicted < 40%	80 (40/40)	Baduanjin	Breathing + walking exercises	6	④
Cao et al., 2022 [21]	Chinese	FEV_1_% < 80%	60 (29/31)	Liuzijue	Conventional	6	①③
Chan et al., 2013 [22]	English	Mean FEV_1_% predicted 53.8%	137 (70/67)	Tai Chi	Usual medical treatment	6	①②④
Chen, 2020 [23]	Chinese	6.67 ± 1.41	120 (60/60)	Baduanjin	Breathing exercises	6	①②③
Chen et al., 2008 [24]	Chinese	Stable stage	40 (21/19)	Liuzijue	Routine health guidance	3	①③
Chen et al., 2009 [25]	Chinese	Grade: II~III	60 (31/29)	Liuzijue	Conventional	3	①③④
Deng & Chen, 2015 [26]	Chinese	Grade: II~IV	61 (31/30)	Baduanjin	Conventional treatment and health education	3	①②③
Deng et al., 2018 [27]	Chinese	12.05 ± 7.57	54 (28/26)	Liuzijue	Breathing exercises	3	④
Fan et al., 2023 [28]	Chinese	10.13 ± 1.96	60 (30/30)	Liuzijue	Resistance training	6	①②③④
Fang et al., 2022 [29]	Chinese	1.27 ± 0.28	60 (30/30)	Liuzijue	Conventional	3	④
Feng, 2019 [30]	Chinese	15.46 ± 4.73	84 (42/42)	Liuzijue	Conventional	3	①②③
He, 2018 [31]	Chinese	13.07 ± 3.45	84 (42/42)	Baduanjin	Conventional	1	④
Hu et al., 2021 [32]	Chinese	FEV_1_ predicted 30~70%	38 (18/20)	Liuzijue	Conventional	6	①③④
Huang & Gao, 2016 [33]	Chinese	Grade: III~IV	61 (31/30)	Baduanjin	Conventional	3	①②③
Huang et al., 2017 [34]	Chinese	2.05 ± 0.89	62 (31/31)	Baduanjin	Conventional	6	①③
Ju et al., 2022 [35]	Chinese	13.66 ± 4.91	160 (80/80)	Liuzijue	Conventional	6	①②③④
Li et al., 2012 [36]	Chinese	Grade: II~III	60 (30/30)	Tai Chi	Breathing exercises	6	①②③
Liu et al., 2019 [37]	Chinese	16.73 ± 8.75	80 (40/40)	Liuzijue	Conventional	3	③
Luo et al., 2023 [38]	English	FEV_1_/FVC < 0.7	226 (116/110)	Tai Chi	Conventional	12	①②③
Ma et al., 2022 [39]	Chinese	3.34 ± 0.49	82 (41/41)	Baduanjin	Pulmonary rehabilitation training	3	①②④
Ma et al., 2025 [40]	Chinese	12.32 ± 4.03	60 (30/30)	Baduanjin	Conventional	3	①③④
Qian & Lin, 2019 [41]	Chinese	9.71 ± 1.92	112 (56/56)	Baduanjin	Conventional	9	①②
Shen, 2017 [42]	Chinese	FEV_1_/FVC < 70%	92 (45/47)	Liuzijue	Health education	6	①②③
Wang & Fang, 2018 [43]	Chinese	15.17 ± 6.73	73 (37/36)	Baduanjin	Conventional basic treatment and health education	3	①②③④
Wang et al., 2019 [44]	English	7.60 ± 7.75	50 (26/24)	Tai Chi	Conventional	3	①③④
Wu et al., 2021 [45]	Chinese	8.19 ± 2.24	103 (51/52)	Baduanjin	Respiratory rehabilitation training	6	①②
Wu et al., 2023 [46]	Chinese	5.46 ± 1.04	110 (55/55)	Liuzijue	Respiratory rehabilitation training	3	①②③④
Xiao et al., 2015 [47]	English	FEV_1_ predicted < 70%	126 (63/63)	Liuzijue	Walking exercise	6	④
Lan et al., 2016 [48]	Chinese	8.21 ± 2.83	84 (42/42)	Liuzijue	Tiotropium bromide alone	3	③
Zhang, 2019 [49]	Chinese	NA	120 (60/60)	Liuzijue	Respiratory rehabilitation training	3	①④
Zhang et al., 2012 [50]	Chinese	NA	60 (30/30)	Tai Chi	Respiratory rehabilitation training	12	①③④
Zhang et al., 2014 [51]	Chinese	33.41 ± 2.45	36 (18/18)	Tai Chi	Conventional	12	①③④
Zhang et al., 2025 [52]	Chinese	NA	108 (55/53)	Baduanjin	Conventional	6	④
Zheng et al., 2022 [53]	English	Grade: II~III	100 (50/50)	Liuzijue	Conventional	1	①②③
Zhu et al., 2018 [54]	English	Grade: II~IV	52 (25/27)	Tai Chi	Usual care with self-management	9	④

Note: NA = not reported; ① = FEV_1_, ② = FVC, ③ FEV_1_/FVC, ④ = 6MWD.

**Table 2:** Pooled Effects and Heterogeneity for the Main Outcomes.

Outcome	k/Studies	Effect Size	95% CI	95% PI	I2
Overall pulmonary function	65/28	g = 0.62	0.40–0.83	−0.61–1.84	91.0%
FEV_1_	26/26	g = 0.71	0.40–1.02	—	—
FEV_1_ (raw scale)	26/26	MD = 0.22 L	0.15–0.29 L	−0.10–0.54 L	—
FVC	16/16	g = 0.52	0.28–0.76	—	—
FVC (raw scale)	16/16	MD = 0.28 L	0.15–0.40 L	−0.15–0.71 L	—
FEV_1_/FVC	23/23	g = 0.53	0.25–0.81	—	—
FEV_1_/FVC (raw scale)	23/23	MD = 3.35 percentage points	1.66–5.05	−3.73–10.43	—
6MWD (standardized)	20/20	g = 0.78	0.57–0.99	−0.02–1.57	71.0%
6MWD (raw scale)	20/20	MD = 34.07 m	25.88–42.25 m	7.53–60.60 m	68.7%

Note: —, not reported for the corresponding outcome-specific analysis.

## Data Availability

The data supporting the findings of this study are provided in the article and its Supplementary Materials. Additional extracted data and statistical analysis code are available from the corresponding author upon reasonable request.

## Supplementary Materials

The following supporting information can be downloaded at https://www.mdpi.com/article/10.3390/life16091444/s1. Table S1. Complete database search strategies and search yields; Table S2. Subgroup pooled effects and moderator tests; Table S3. Continuous intervention-duration meta-regression and model fit; Table S4. Exploratory small-study effect diagnostics; Table S5. Sensitivity analyses; Figure S1. Complete effect-level forest plot for pulmonary function; Figure S2. Funnel plots and trim-and-fill analyses; Figure S3. Sensitivity analyses for pulmonary function; Figure S4. Sensitivity analyses for the raw mean difference in 6MWD.
