# Supplementary material for: Effects of Mind–Body Exercise on Pulmonary Function and Functional Exercise Capacity in Older Adults with COPD: A Three-Level Meta-Analysis
Source: Life (Basel). 2026 Aug 30;16(9):1444. doi: 10.3390/life16091444 (PMC13608156; doi:10.3390/life16091444)
Supplement: Supplementary file 1 [file life-16-01444-s001.zip › Supplementary_Figure_S1_Complete effect-level forest plot for pulmonary function.pdf]

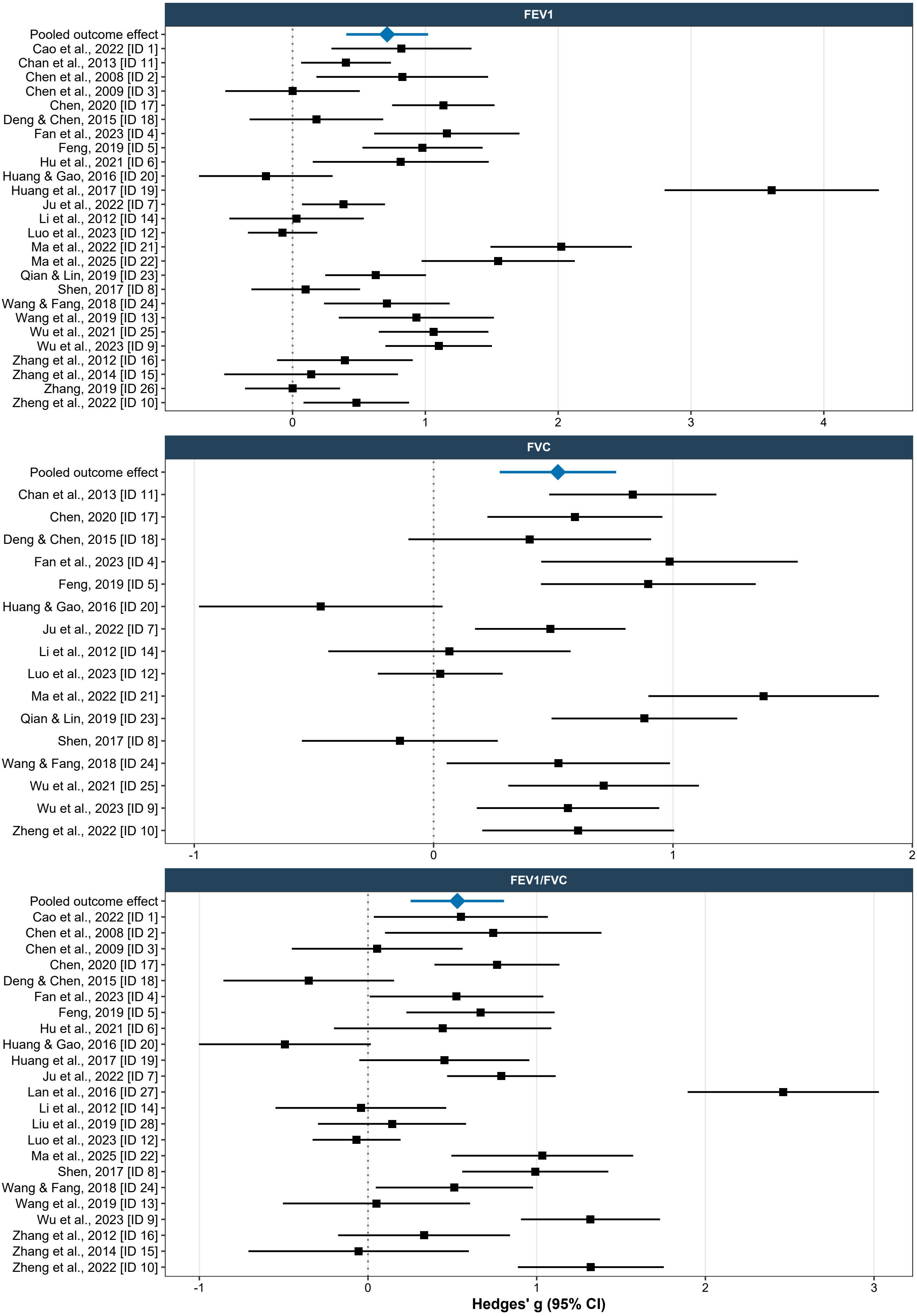
FEV1
Pooled outcome effect
Cao et al., 2022 [ID 1]
Chan et al., 2013 [ID 11]
Chen et al., 2008 [ID 2]
Chen et al., 2009 [ID 3]
Chen, 2020 [ID 17]
Deng & Chen, 2015 [ID 18]
Fan et al., 2023 [ID 4]
Feng, 2019 [ID 5]
Hu et al., 2021 [ID 6]
Huang & Gao, 2016 [ID 20]
Huang et al., 2017 [ID 19]
Ju et al., 2022 [ID 7]
Li et al., 2012 [ID 14]
Luo et al., 2023 [ID 12]
Ma et al., 2022 [ID 21]
Ma et al., 2025 [ID 22]
Qian & Lin, 2019 [ID 23]
Shen, 2017 [ID 8]
Wang & Fang, 2018 [ID 24]
Wang et al., 2019 [ID 13]
Wu et al., 2021 [ID 25]
Wu et al., 2023 [ID 9]
Zhang et al., 2012 [ID 16]
Zhang et al., 2014 [ID 15]
Zhang, 2019 [ID 26]
Zheng et al., 2022 [ID 10]
0
1
2
3
4
FVC
Pooled outcome effect
Chan et al., 2013 [ID 11]
Chen, 2020 [ID 17]
Deng & Chen, 2015 [ID 18]
Fan et al., 2023 [ID 4]
Feng, 2019 [ID 5]
Huang & Gao, 2016 [ID 20]
Ju et al., 2022 [ID 7]
Li et al., 2012 [ID 14]
Luo et al., 2023 [ID 12]
Ma et al., 2022 [ID 21]
Qian & Lin, 2019 [ID 23]
Shen, 2017 [ID 8]
Wang & Fang, 2018 [ID 24]
Wu et al., 2021 [ID 25]
Wu et al., 2023 [ID 9]
Zheng et al., 2022 [ID 10]
-1
0
1
2
FEV1/FVC
Pooled outcome effect
Cao et al., 2022 [ID 1]
Chen et al., 2008 [ID 2]
Chen et al., 2009 [ID 3]
Chen, 2020 [ID 17]
Deng & Chen, 2015 [ID 18]
Fan et al., 2023 [ID 4]
Feng, 2019 [ID 5]
Hu et al., 2021 [ID 6]
Huang & Gao, 2016 [ID 20]
Huang et al., 2017 [ID 19]
Ju et al., 2022 [ID 7]
Lan et al., 2016 [ID 27]
Li et al., 2012 [ID 14]
Liu et al., 2019 [ID 28]
Luo et al., 2023 [ID 12]
Ma et al., 2025 [ID 22]
Shen, 2017 [ID 8]
Wang & Fang, 2018 [ID 24]
Wang et al., 2019 [ID 13]
Wu et al., 2023 [ID 9]
Zhang et al., 2012 [ID 16]
Zhang et al., 2014 [ID 15]
Zheng et al., 2022 [ID 10]
-1
0
1
2
3
Hedges' g (95% CI)
