# Supplementary material for: Effects of Mind–Body Exercise on Pulmonary Function and Functional Exercise Capacity in Older Adults with COPD: A Three-Level Meta-Analysis
Source: Life (Basel). 2026 Aug 30;16(9):1444. doi: 10.3390/life16091444 (PMC13608156; doi:10.3390/life16091444)
Supplement: Supplementary file 1 [file life-16-01444-s001.zip › Supplementary_Figure_S2_Funnel plots and trim-and-fill analyses.pdf]

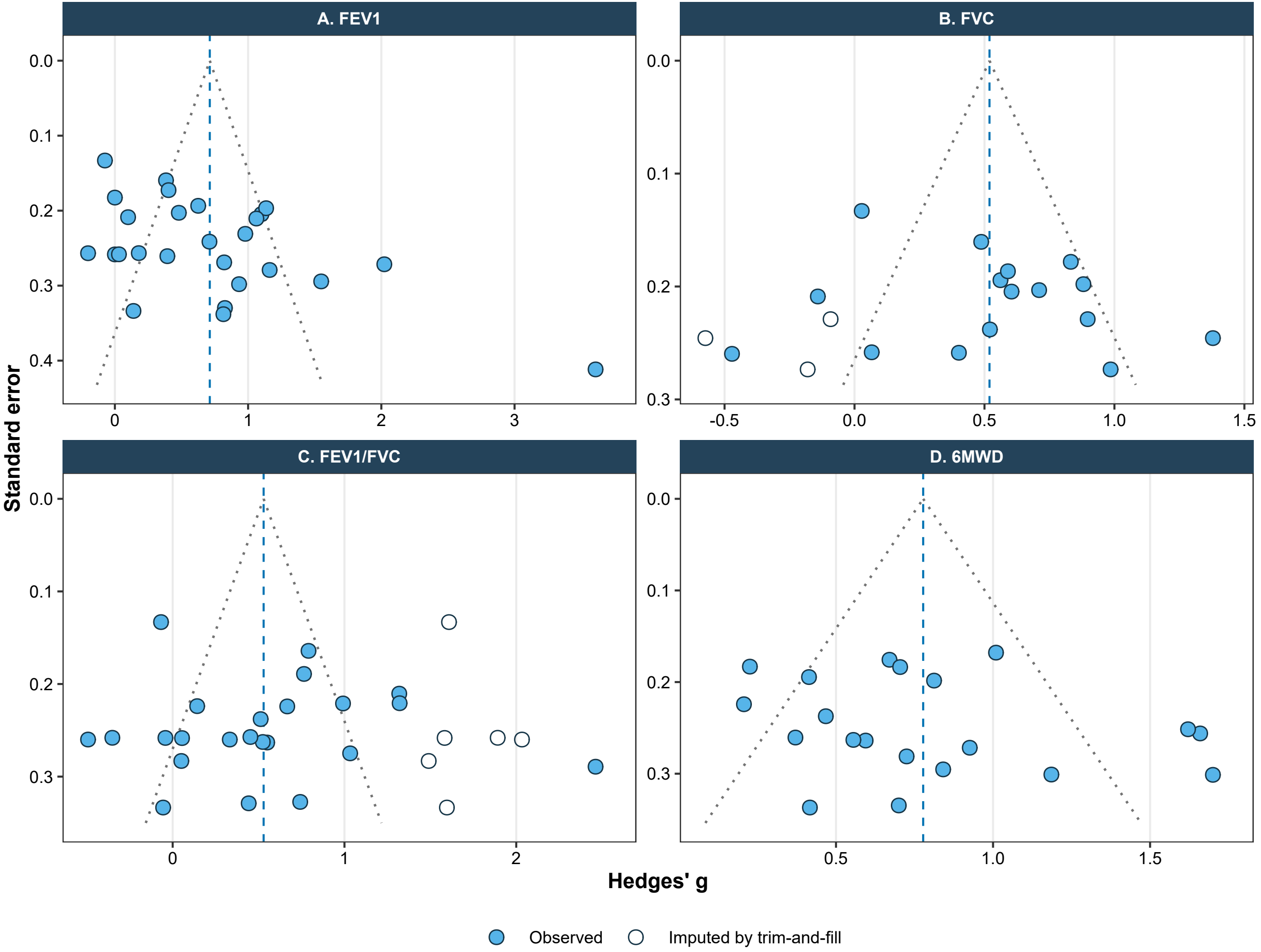
A. FEV1
B. FVC
C. FEV1/FVC
D. 6MWD
Standard error
Hedges' g
0.0
0.1
0.2
0.3
0.4
0
1
2
3
-0.5
0.0
0.5
1.0
1.5
Observed
Imputed by trim-and-fill
