# Supplementary material for: Effects of Mind–Body Exercise on Pulmonary Function and Functional Exercise Capacity in Older Adults with COPD: A Three-Level Meta-Analysis
Source: Life (Basel). 2026 Aug 30;16(9):1444. doi: 10.3390/life16091444 (PMC13608156; doi:10.3390/life16091444)
Supplement: Supplementary file 1 [file life-16-01444-s001.zip › Supplementary_Figure_S3_Sensitivity analyses for pulmonary function.pdf]

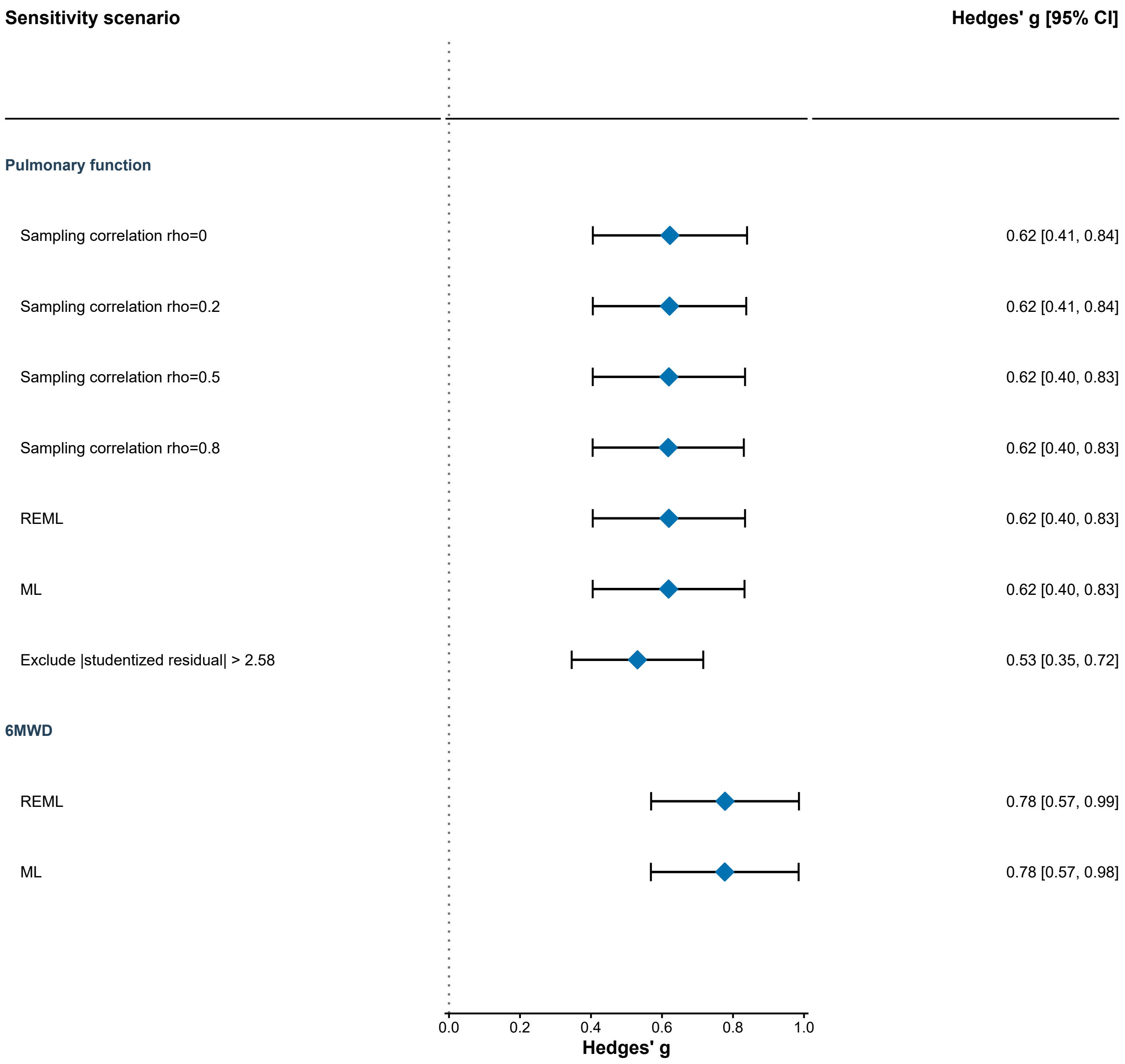
Sensitivity scenario
Hedges' g [95% CI]
Pulmonary function
Sampling correlation rho=0
0.62 [0.41, 0.84]
Sampling correlation rho=0.2
0.62 [0.41, 0.84]
Sampling correlation rho=0.5
0.62 [0.40, 0.83]
Sampling correlation rho=0.8
0.62 [0.40, 0.83]
REML
0.62 [0.40, 0.83]
ML
0.62 [0.40, 0.83]
Exclude |studentized residual| > 2.58
0.53 [0.35, 0.72]
6MWD
REML
0.78 [0.57, 0.99]
ML
0.78 [0.57, 0.98]
0.0
0.2
0.4
0.6
0.8
1.0
Hedges' g
