# Supplementary material for: Effects of Mind–Body Exercise on Pulmonary Function and Functional Exercise Capacity in Older Adults with COPD: A Three-Level Meta-Analysis
Source: Life (Basel). 2026 Aug 30;16(9):1444. doi: 10.3390/life16091444 (PMC13608156; doi:10.3390/life16091444)
Supplement: Supplementary file 1 [file life-16-01444-s001.zip › Supplementary_Figure_S4_Sensitivity analyses for the raw mean difference in 6MWD.pdf]

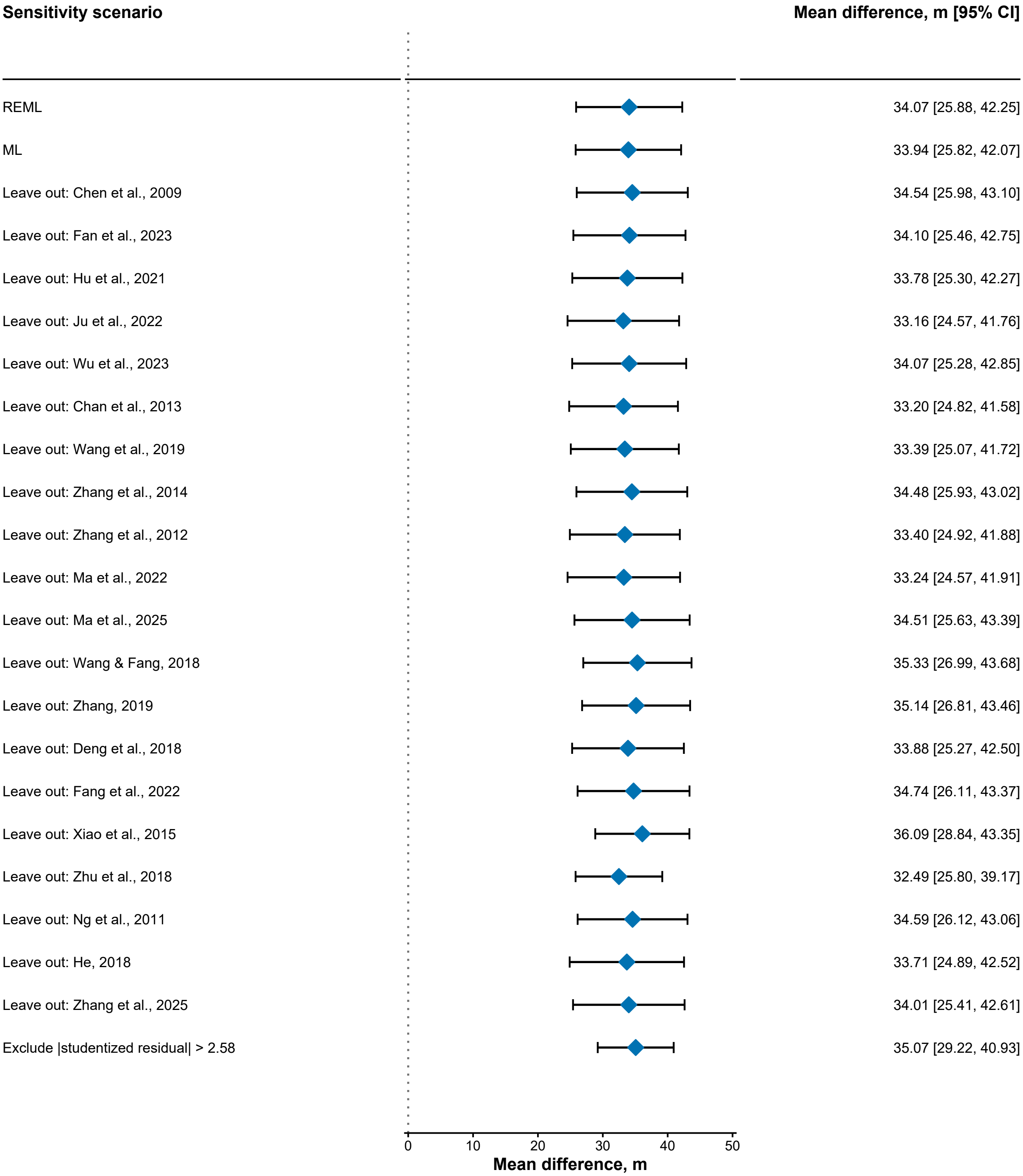

Sensitivity scenario
Mean difference, m [95% CI]
REML
34.07 [25.88, 42.25]
ML
33.94 [25.82, 42.07]
Leave out: Chen et al., 2009
34.54 [25.98, 43.10]
Leave out: Fan et al., 2023
34.10 [25.46, 42.75]
Leave out: Hu et al., 2021
33.78 [25.30, 42.27]
Leave out: Ju et al., 2022
33.16 [24.57, 41.76]
Leave out: Wu et al., 2023
34.07 [25.28, 42.85]
Leave out: Chan et al., 2013
33.20 [24.82, 41.58]
Leave out: Wang et al., 2019
33.39 [25.07, 41.72]
Leave out: Zhang et al., 2014
34.48 [25.93, 43.02]
Leave out: Zhang et al., 2012
33.40 [24.92, 41.88]
Leave out: Ma et al., 2022
33.24 [24.57, 41.91]
Leave out: Ma et al., 2025
34.51 [25.63, 43.39]
Leave out: Wang & Fang, 2018
35.33 [26.99, 43.68]
Leave out: Zhang, 2019
35.14 [26.81, 43.46]
Leave out: Deng et al., 2018
33.88 [25.27, 42.50]
Leave out: Fang et al., 2022
34.74 [26.11, 43.37]
Leave out: Xiao et al., 2015
36.09 [28.84, 43.35]
Leave out: Zhu et al., 2018
32.49 [25.80, 39.17]
Leave out: Ng et al., 2011
34.59 [26.12, 43.06]
Leave out: He, 2018
33.71 [24.89, 42.52]
Leave out: Zhang et al., 2025
34.01 [25.41, 42.61]
Exclude |studentized residual| > 2.58
35.07 [29.22, 40.93]
0
10
20
30
40
50
Mean difference, m
