# Supplementary material for: Effects of Mind–Body Exercise on Pulmonary Function and Functional Exercise Capacity in Older Adults with COPD: A Three-Level Meta-Analysis
Source: Life (Basel). 2026 Aug 30;16(9):1444. doi: 10.3390/life16091444 (PMC13608156; doi:10.3390/life16091444)
Supplement: Supplementary file 1 [file life-16-01444-s001.zip › Supplementary_Tables_S1-S5.pdf]

Supplementary Table S1. Complete database search strategies and search yields

Search period: from database inception to June 2026.

Database	Search strategy	Records retrieved, n
PubMed	(("Pulmonary Disease, Chronic Obstructive"[Mesh] OR COPD OR "Chronic Obstructive Lung Disease" OR "Chronic Airflow Obstruction") AND ("Tai Ji"[Mesh] OR "Tai Chi" OR Taiji OR "Baduanjin" OR "Liuzijue" OR "Qigong" OR "Qi Gong" OR "Yoga"[Mesh] OR Yoga OR "Mind-Body Therapies"[Mesh] OR "Mind Body Exercise")) AND ("Randomized Controlled Trial"[Publication Type] OR randomized controlled trial OR randomized OR randomised OR RCT)	337
Web of Science Core Collection	("chronic obstructive pulmonary disease" OR COPD OR "chronic obstructive lung disease" OR "chronic airflow obstruction") AND ("Tai Chi" OR "Tai Ji" OR Taiji OR "Baduanjin" OR "Liuzijue" OR "Qigong" OR "Qi Gong" OR "Yoga" OR "Mind-Body Exercise" OR "Mind-Body Therapies") AND ("randomized controlled trial" OR RCT OR randomized OR randomised)	182
Embase	('chronic obstructive lung disease'/exp OR COPD:ti,ab,kw OR 'chronic obstructive pulmonary disease':ti,ab,kw) AND ('tai chi'/exp OR 'baduanjin':ti,ab,kw OR 'liuzijue':ti,ab,kw OR 'qigong'/exp OR 'yoga'/exp OR 'mind body exercise':ti,ab,kw) AND ('randomized controlled trial'/exp OR randomized:ti,ab,kw OR RCT:ti,ab,kw)	202
Cochrane Library	("chronic obstructive pulmonary disease" OR COPD OR "chronic obstructive lung disease") AND ("Tai Chi" OR "Tai Ji" OR Taiji OR "Baduanjin" OR "Liuzijue" OR "Qigong" OR "Qi Gong" OR "Yoga" OR "Mind-Body Exercise" OR "Mind-Body Therapies") AND ("randomized controlled trial" OR randomized OR randomised OR RCT)	179
CNKI	主题 = ("慢性阻塞性肺疾病" OR "慢阻肺" OR COPD) AND 主题 = ("太极拳" OR "八段锦" OR "六字诀" OR "气功" OR "瑜伽" OR "身心运动")	352
Wanfang	主题 = ("慢性阻塞性肺疾病" OR "慢阻肺" OR COPD) AND 主题 = ("太极拳" OR "八段锦" OR "六字诀" OR "气功" OR "瑜伽" OR "身心运动")	229
	**Total records identified**	**1,481**

Notes: CNKI, China National Knowledge Infrastructure; COPD, chronic obstructive pulmonary disease; RCT, randomized controlled trial. Search strings are reproduced from the recorded search log; database names, spacing, and quotation-mark typography were standardized without changing the Boolean logic.

Table S2. Subgroup pooled effects and omnibus moderator tests

Panel A. Subgroup-specific pooled effects

Domain	Moderator	Subgroup	k	Studies	Hedges' g	95% CI	95% PI	p (subgroup)	Inference
Pulmonary function	Pulmonary outcome	FEV1	26	26	0.71	0.40 to 1.02	-0.74 to 2.16	<0.001	Knapp-Hartung
Pulmonary function	Pulmonary outcome	FVC	16	16	0.52	0.28 to 0.76	-0.35 to 1.39	<0.001	Knapp-Hartung
Pulmonary function	Pulmonary outcome	FEV1/FVC	23	23	0.53	0.25 to 0.81	-0.71 to 1.77	<0.001	Knapp-Hartung
Pulmonary function	Exercise modality	Baduanjin	22	9	0.80	0.26 to 1.34	-1.14 to 2.74	0.010	CR2 cluster-robust
Pulmonary function	Exercise modality	Liuzijue	29	13	0.68	0.38 to 0.98	-0.49 to 1.85	<0.001	CR2 cluster-robust
Pulmonary function	Exercise modality	Tai Chi	14	6	0.24	-0.10 to 0.57	-0.50 to 0.97	0.131	CR2 cluster-robust
Pulmonary function	Intervention duration	≤3 months	31	14	0.68	0.27 to 1.10	-0.91 to 2.27	0.004	CR2 cluster-robust
Pulmonary function	Intervention duration	>3 to <12 months	27	11	0.68	0.42 to 0.94	-0.51 to 1.87	<0.001	CR2 cluster-robust
Pulmonary function	Intervention duration	≥12 months	7	3	0.07	-0.66 to 0.80	-0.71 to 0.86	0.641	CR2 cluster-robust
6MWD	Exercise modality	Baduanjin	6	6	1.00	0.24 to 1.75	-0.89 to 2.88	0.019	Knapp-Hartung
6MWD	Exercise modality	Liuzijue	9	9	0.64	0.45 to 0.84	0.19 to 1.10	<0.001	Knapp-Hartung
6MWD	Exercise modality	Tai Chi	5	5	0.79	0.48 to 1.09	0.48 to 1.09	0.002	Knapp-Hartung
6MWD	Intervention duration	≤3 months	10	10	0.88	0.48 to 1.28	-0.31 to 2.08	<0.001	Knapp-Hartung
6MWD	Intervention duration	>3 to <12 months	8	8	0.68	0.43 to 0.93	0.14 to 1.21	<0.001	Knapp-Hartung
6MWD	Intervention duration	≥12 months	2	2	0.71	-2.48 to 3.91	-3.29 to 4.71	0.217	Knapp-Hartung

Panel B. Omnibus moderator tests with multiplicity adjustment

Domain	Moderator	Test statistic	df1	df2	Unadjusted p	Holm-adjusted p	BH-FDR q	Inference
Pulmonary function	Pulmonary outcome	1.835	2	19.23	0.187	0.560	0.311	CR2 HTZ joint Wald test
Pulmonary function	Exercise modality	3.832	2	13.51	0.048	0.240	0.121	CR2 HTZ joint Wald test
Pulmonary function	Intervention duration	5.543	2	5.48	0.048	0.240	0.121	CR2 HTZ joint Wald test
6MWD	Exercise modality	1.049	2	17.00	0.372	0.744	0.465	Model-based omnibus test
6MWD	Intervention duration	0.463	2	17.00	0.637	0.744	0.637	Model-based omnibus test

Notes: Positive Hedges' g favors mind-body exercise. Subgroup estimates are between-study summaries and should not be interpreted as head-to-head comparisons. The five omnibus moderator p values were adjusted jointly using the Holm method; Benjamini–Hochberg false-discovery-rate (BH-FDR) q values are also shown. Prediction intervals are model-based.

Table S3. Continuous intervention-duration meta-regression and model fit

Panel A. REML meta-regression coefficients

Domain	Model	Term	β	SE	95% CI	p	Holm-adjusted p	BH-FDR q	Inference
Pulmonary function	Linear	Intercept	0.583	0.093	0.387 to 0.779	<0.001	—	—	CR2 cluster-robust
Pulmonary function	Linear	Duration (centered at 6 months)	-0.047	0.028	-0.114 to 0.020	0.142	0.852	0.366	CR2 cluster-robust
Pulmonary function	Quadratic	Intercept	0.685	0.114	0.435 to 0.935	<0.001	—	—	CR2 cluster-robust
Pulmonary function	Quadratic	Duration (centered at 6 months)	-0.031	0.033	-0.104 to 0.043	0.377	0.979	0.443	CR2 cluster-robust
Pulmonary function	Quadratic	Duration²	-0.010	0.007	-0.025 to 0.006	0.199	0.979	0.366	CR2 cluster-robust
6MWD	Linear	Intercept	0.752	0.106	0.529 to 0.974	<0.001	—	—	Knapp-Hartung
6MWD	Linear	Duration (centered at 6 months)	-0.028	0.036	-0.103 to 0.047	0.443	0.979	0.443	Knapp-Hartung
6MWD	Quadratic	Intercept	0.616	0.144	0.311 to 0.920	<0.001	—	—	Knapp-Hartung
6MWD	Quadratic	Duration (centered at 6 months)	-0.045	0.037	-0.124 to 0.034	0.244	0.979	0.366	Knapp-Hartung
6MWD	Quadratic	Duration²	0.014	0.010	-0.008 to 0.035	0.196	0.979	0.366	Knapp-Hartung

Panel B. ML model-fit comparison

Domain	Model	AIC	BIC	ΔAIC	ΔBIC	Log likelihood
Pulmonary function	Linear	122.721	131.419	0.000	0.000	-57.361
Pulmonary function	Quadratic	123.785	134.657	1.064	3.238	-56.893
6MWD	Linear	28.817	31.804	0.047	0.000	-11.408
6MWD	Quadratic	28.769	32.752	0.000	0.948	-10.385

Notes: Intervention duration was centered at 6 months. Final regression coefficients were estimated using REML; ML was used only for AIC/BIC comparison of linear versus quadratic fixed-effect structures. Holm and BH-FDR adjustments apply jointly to the six non-intercept duration terms. No adjusted duration term was statistically significant.

Table S4. Exploratory small-study effect diagnostics

Outcome	k	Pooled g (95% CI)	Egger statistic	Egger p	Begg τ	Begg p	Trim-and-fill k_0	Adjusted g (95% CI)
FEV1	26	0.71 (0.43 to 0.99)	3.066	0.002	0.268	0.057	0	0.71 (0.43 to 0.99)
FVC	16	0.52 (0.30 to 0.74)	0.259	0.796	-0.017	0.965	3	0.40 (0.17 to 0.63)
FEV1/FVC	23	0.53 (0.27 to 0.79)	-0.126	0.899	-0.083	0.601	6	0.77 (0.50 to 1.04)
6MWD	20	0.78 (0.58 to 0.97)	1.069	0.285	0.189	0.260	0	0.78 (0.58 to 0.97)

Notes: Egger regression, Begg rank correlation, and trim-and-fill were treated as exploratory diagnostics of small-study effects. Funnel-plot asymmetry can arise from heterogeneity and design differences and should not be interpreted as definitive evidence of publication bias. Trim-and-fill estimates are shown for descriptive sensitivity only. Pooled and trim-and-fill estimates in this table were obtained from outcome-specific two-level models used solely for small-study-effect diagnostics and may therefore differ slightly from the primary or subgroup estimates.

Table S5. Sensitivity analyses

Panel A. Primary effect sensitivity analyses

Domain	Sensitivity analysis	Effect measure	Estimate	95% CI	95% PI	k	Studies	Inference	Model
Pulmonary function	Assumed sampling correlation rho=0	Hedges' g	0.62	0.41 to 0.84	-0.60 to 1.84	65	28	CR2 cluster-robust	Three-level model
Pulmonary function	Assumed sampling correlation rho=0.2	Hedges' g	0.62	0.41 to 0.84	-0.60 to 1.84	65	28	CR2 cluster-robust	Three-level model
Pulmonary function	Assumed sampling correlation rho=0.5	Hedges' g	0.62	0.40 to 0.83	-0.61 to 1.84	65	28	CR2 cluster-robust	Three-level model
Pulmonary function	Assumed sampling correlation rho=0.8	Hedges' g	0.62	0.40 to 0.83	-0.61 to 1.85	65	28	CR2 cluster-robust	Three-level model
Pulmonary function	REML	Hedges' g	0.62	0.40 to 0.83	-0.61 to 1.84	65	28	CR2 cluster-robust	Three-level model
Pulmonary function	ML	Hedges' g	0.62	0.40 to 0.83	-0.58 to 1.82	65	28	CR2 cluster-robust	Three-level model
6MWD	REML	Hedges' g	0.78	0.57 to 0.99	-0.02 to 1.57	20	20	Knapp-Hartung	Two-level model
6MWD	ML	Hedges' g	0.78	0.57 to 0.98	0.01 to 1.54	20	20	Knapp-Hartung	Two-level model
Pulmonary function	Exclude studies containing |studentized residual| > 2.58	Hedges' g	0.53	0.35 to 0.72	-0.42 to 1.48	62	26	CR2 cluster-robust	Three-level model
6MWD	REML	Mean difference (metres)	34.07	25.88 to 42.25	7.53 to 60.60	20	20	Knapp-Hartung	Two-level model
6MWD	ML	Mean difference (metres)	33.94	25.82 to 42.07	8.50 to 59.39	20	20	Knapp-Hartung	Two-level model
6MWD	Exclude studies containing |studentized residual| > 2.58	Mean difference (metres)	35.07	29.22 to 40.93	19.99 to 50.15	18	18	Knapp-Hartung	Two-level model

Panel B. Pulmonary heterogeneity across assumed within-study sampling correlations

ρ	τ^2 between	τ^2 within	I^2 between (%)	I^2 within (%)	I^2 total (%)	Q_E	Q_E p
0.0	0.235	0.106	60.2	27.2	87.4	406.93	<0.001
0.2	0.217	0.126	55.8	32.3	88.1	368.76	<0.001
0.5	0.189	0.156	49.9	41.1	91.0	388.07	<0.001
0.8	0.161	0.188	44.2	51.6	95.8	635.52	<0.001

Panel C. 6MWD raw mean-difference leave-one-study-out analysis

Omitted study	Pooled MD, m	SE	95% CI	p	k	Studies
Chen et al 2009	34.54	4.07	25.98 to 43.10	<0.001	19	19
Fan et al 2023	34.10	4.11	25.46 to 42.75	<0.001	19	19
Hu et al 2021	33.78	4.04	25.30 to 42.27	<0.001	19	19
Ju et al 2022	33.16	4.09	24.57 to 41.76	<0.001	19	19
Wu et al 2023	34.07	4.18	25.28 to 42.85	<0.001	19	19
Chan et al 2013	33.20	3.99	24.82 to 41.58	<0.001	19	19
Wang et al 2019	33.39	3.96	25.07 to 41.72	<0.001	19	19
Zhang et al 2014	34.48	4.07	25.93 to 43.02	<0.001	19	19
Zhang et al 2012	33.40	4.04	24.92 to 41.88	<0.001	19	19
Ma et al 2022	33.24	4.13	24.57 to 41.91	<0.001	19	19
Ma et al 2025	34.51	4.23	25.63 to 43.39	<0.001	19	19
Wang & Fang 2018	35.33	3.97	26.99 to 43.68	<0.001	19	19
Zhang 2019	35.14	3.96	26.81 to 43.46	<0.001	19	19
Deng et al 2018	33.88	4.10	25.27 to 42.50	<0.001	19	19
Fang et al 2022	34.74	4.11	26.11 to 43.37	<0.001	19	19
Xiao et al 2015	36.09	3.45	28.84 to 43.35	<0.001	19	19
Zhu et al 2018	32.49	3.18	25.80 to 39.17	<0.001	19	19
Ng et al 2011	34.59	4.03	26.12 to 43.06	<0.001	19	19
He 2018	33.71	4.20	24.89 to 42.52	<0.001	19	19
Zhang et al 2025	34.01	4.09	25.41 to 42.61	<0.001	19	19

Notes: The leave-one-study-out pooled 6MWD mean difference ranged from 32.49 to 36.09 m. The pulmonary primary model used ρ=0.50 and REML with study-clustered CR2 inference. Changes in the pulmonary variance split across ρ values should not be interpreted mechanistically.
